# Pharmacokinetic comparison of a diverse panel of non-targeting human antibodies as matched IgG1 and IgG2 isotypes in rodents and non-human primates

**DOI:** 10.1371/journal.pone.0217061

**Published:** 2019-05-23

**Authors:** Kenneth W. Walker, Hossein Salimi-Moosavi, Gregory E. Arnold, Qing Chen, Marcus Soto, Frederick W. Jacobsen, John Hui

**Affiliations:** Amgen Research, Amgen Inc., One Amgen Center Drive, Thousand Oaks, CA, United States of America; King's College London, UNITED KINGDOM

## Abstract

In this study we compared the pharmacokinetic profile of four unrelated antibodies, which do not bind to mammalian antigens, in IgG1 and IgG2 frameworks in both rats and non-human primates (NHP). This allowed for extensive cross comparison of the impact of antibody isotype, complementarity determining regions (CDR) and model species on pharmacokinetics without the confounding influence of antigen binding in the hosts. While antibody isotype had no significant impact on the pharmacokinetics, the CDRs do alter the profile, and there is an inverse correlation between the neonatal Fc receptor (FcRn) affinity and pharmacokinetic performance. Faster clearance rates were also associated with higher isoelectric points; however, although this panel of antibodies all possess basic isoelectric points, ranging from 8.44 to 9.18, they also have exceptional *in vivo* half-lives, averaging 369 hours, and low clearance rates, averaging 0.18 ml/h/kg in NHPs. This pattern of pharmacokinetic characteristics was conserved between rats and NHPs.

## Introduction

Human IgG1 antibodies are frequently employed as human therapeutics, whereas human IgG2, IgG4 [1] and other antibody subclasses are less commonly used [1, 2, 3, 4, 5, 6, 7, 8, 9, 10]. While rodents are heavily utilized for pharmacokinetic (PK) assessment of antibodies, non-human primates, most commonly NHPs, are generally regarded as the better predictors of human pharmacokinetics for these molecules [11]. Even though human IgG1 antibodies all possess a high level of identity due to their shared constant domains and variable domain framework, the PK attributes of different antibodies can vary widely. For example, the PK attributes of approved therapeutic antibodies in humans have a wide range with reported systemic half-lives from 79 to 648 hours and clearance rates of 0.141 to 1.017 ml/h/kg [12, 13]. The PK of conventional antibodies in NHPs also varies widely with serum half-lives ranging from 29 to 299 hours and clearance rates of 0.07 to 1.14 (ml/h/kg) [2, 3, 4, 5, 6, 7, 8].

There are many factors that can impact the PK of antibodies including antibody susceptibility to degradation pathways, charge characteristics, glycosylation patterns, neonatal Fc receptor (FcRn) recycling efficiency, target mediated clearance, IgG isotype, and anti-drug antibody responses (ADA) [14, 15, 16, 17, 18, 19, 20, 21, 22, 23, 24]. Since FcRn mediated antibody recycling is primarily responsible for the exceptional *in vivo* half-life of antibodies, this interaction has been a major focus of engineering efforts to further enhance the PK of therapeutic antibodies [5, 6, 25, 26, 27]. Mutations in the CH2 and CH3 domains that modulate FcRn interactions show extended half-lives in NHPs ranging from 113 to 746 hours [5, 6, 7, 28, 29], which is up to a 3-fold improvement. In contrast, target mediated clearance, whereby an antibody is removed from circulation by its ligand, can have a substantial negative impact on antibody PK, particularly at lower dose levels, and it is most commonly observed as non-linear dose-dependent PK [30, 31, 32, 33, 34, 35, 36]. For example, antibodies that bind to cell surface receptors are often internalized and ultimately digested in the lysosome, resulting in a saturable elimination mechanism for that antibody. Similarly, anti-drug antibody (ADA) responses, whereby the host develops antibodies targeting the therapeutic antibody, ultimately leading to its degradation, can also lead to rapid clearance of therapeutic antibodies [37, 38, 39]. However, unlike target mediated clearance that displays a dose response shift in PK, ADA responses typically do not manifest until seven or more days after the first administration of the therapeutic antibody due to the time it takes to illicit the host immune response, at which point the serum levels can substantially deviate from a standard PK profile [40]. It has also been proposed that antibodies with lower isoelectric points (pI) have better PK than those with higher pIs [41]; however, others have reported that changing the pI up or down can both decrease antibody residence *in vivo* [42] or have no measurable impact at all [43].

Here we examine the pharmacokinetic behavior of a panel of four unrelated antibodies that do not bind to mammalian ligands, thus eliminating the confounding factor of target mediated clearance. Furthermore, we examined all four antibodies using both IgG1 and IgG2 frameworks to determine the effect of antibody isotype and variability in the complementarity determining regions (CDRs) on PK. Finally, all eight antibodies were tested in two disparate species, rats and NHPs, to enable a comparison of any species specific impact on PK.

## Materials and methods

### Generation of the antibody panel

Antibody A was generated by immunizing XenoMouse mice using a range of 10–30 μg/mouse of a mammalian protein immunogen emulsified in TiterMax Gold adjuvant (Sigma-Aldrich, Oakville, Ontario) for the initial immunization over a period of 4 weeks. Following the initial immunization, subsequent boost of immunogen (5–20 μg/mouse) were administered on a schedule and for the duration necessary to induce suitable antibodies in the mice. Titers were determined by enzyme immunoassay using immobilized antigen. For antibody A, the parental antibody was then engineered by site-directed mutation of two complementarity determining region (CDR) residues to eliminate binding to the antigen as assessed by surface plasmon resonance (no detectable binding at 30 μM antibody A). Antibodies B & C were also generated by a XenoMouse campaign; however, the antigen used was dinitrophenol (DNP) conjugated to keyhole limpet hemocyanin (KLH), and screening for binders was conducted using DNP conjugated to lysine. Antibody C was engineered for additional stability by incorporating a tryptophan to phenylalanine mutation in one of the CDR residues. Although antibodies B & C both bind DNP, there is no significant sequence relationship between the antibodies. Antibody D was generated utilizing XenoMouse immunization with KLH, and anti-KLH antibodies were identified by screening against immobilized KLH.

### Production of the antibodies

All four recombinant antibodies were expressed using the same proprietary Amgen modified Chinese hamster ovary (CHO) host cell line with expression titers ranging from 0.1 to 2.0 g/l. Antibodies were first purified using MabSelect Sure (GE Healthcare, Piscataway NJ) by directly loading the conditioned media and washing the column with Dulbecco’s phosphate buffered saline (PBS). Antibodies were eluted using 100 mM acetic acid pH 3.2 and the elution pools were brought to pH 5.0 using 1 M NaOH. The MabSelect Sure elution pools were then diluted with two volumes of water and loaded on to a SP-Sepharose HP column (GE Healthcare, Piscataway NJ) followed washing with SP-Buffer A (20 mM acetic acid pH 5.0). The column was then developed using a 20 column volume linear gradient to 100% SP-Buffer B (20 mM acetic acid, 600 mM NaCl, pH 5.0) and fractions were pooled based on Coomassie blue stained SDS-PAGE analysis (64–86% yield). The pooled antibodies were then concentrated and buffer exchanged into 10 mM acetic acid, 9% sucrose, pH 5.2, and aliquots were stored at -70°C until needed. Protein quality was assessed by Coomassie stained sodium dodecyl sulfate polyacrylamide gel electrophoresis (SDS-PAGE), size exclusion high performance liquid chromatography SE-HPLC (BioSep S3000 column, Phenomenex, Torrance, CA, USA) and liquid chromatography electrospray ionization mass-spectrometry.

### *In vivo* rat pharmacokinetic study

Rats were cared for in accordance with the Guide for the Care and Use of Laboratory Animals, 8^th^ Edition. All research protocols were approved by the Amgen, Inc. Institutional Animal Care and Use Committee (protocol number: 2008–00092). Rats were individually housed at an AAALAC, International accredited facility in static caging on corn cob bedding. Rats had *ad libitum* access to pelleted feed (2020X, Harlan, IN, USA) and reverse osmosis-purified water via water bottles. Rats were maintained on a 12:12 hour light:dark cycle in rooms at 64–79°F and humidity range of 30–70%, respectively, and had access to enrichment opportunities (nestlets, Nylabones, and igloos).

The PK profiles of the human IgG1 and IgG2 antibodies were determined in male adult Sprague-Dawley (SD) rats (n = 3 per group, 8 to 12 weeks of age, 300–350 g) by injecting 5 mg/kg subcutaneously (bolus) in the midscapular region of the dorsal back or intravenously and collecting 250 μl samples of blood in conscious animals from the jugular vein catheter or lateral tail vein at 0, 0.25, 1, 4, 24, 48, 72, 168, 336, 504, 672, 840 and 1008 hours post-dose. Each blood sample was maintained at room temperature after collection, and following a 30–40 minute clotting period, samples were centrifuged at 2–8°C at 11,500 rpm for about 10 minutes using a calibrated Eppendorf 5417R Centrifuge System (Brinkmann Instruments, Inc., Westbury, NY). The collected serum was then transferred into a pre-labeled (for each rat), cryogenic storage tube and stored at -60°C to -80°C for future bioanalysis.

### *In vivo* non-human primate pharmacokinetic study

Non-human primates were cared for in accordance with the Guide for the Care and Use of Laboratory Animals, 8^th^ Edition. All research protocols were approved by the MPI Research, Inc. Institutional Animal Care and Use Committee (protocol number: 529–261). Primates were individually housed at an AAALAC, International accredited facility in stainless steel cages. Primates had *ad libitum* access to Lab Diet certified primate diet (#5048, PMI Nutrition, International, Inc., USA) and reverse osmosis-purified water via water bottles. Primates were maintained on a 12:12 hour light:dark cycle in rooms at 64–79°F and humidity range of 30–70%, respectively, and had access to enrichment opportunities (perches, mirrors, and hard plastic toys).

The PK profiles of the human IgG1 and IgG2 antibodies were also determined in Cynomolgus monkeys (referred to as NHPs throughout this manuscript) (n = 2 per group, except A2 for which n = 3) by serial injection of two subsequent subcutaneous bolus doses at 1 mg/kg at mid-scapular region on the dorsal back of animals on day 0 and 5 mg/kg on day 57. The second dose at 5 mg/kg was used to determine the linearity of the PK without using additional animals for the study. Blood samples (approximately 1 ml) were collected in conscious animals from the femoral vein or femoral artery at pre-dose, 0.5, 2, 4, 8, 12, 24, 48, 96, 168, 336, 504, 672, 840, 1008, 1176 and 1344 hours post 1^st^ dose (1 mg/kg) and 0.5, 2, 4, 8, 12, 24, 48, 96, 168, 336, 360, 384, 432, 504, 672, 840, 1008, 1176 and 1344 hours following the second dose (5 mg/kg). The blood samples were allowed to clot at room temperature for at least 30 minutes and then placed on an ice block (or wet ice) until centrifuged. The samples were centrifuged, and the resulting serum was separated, split into approximately three equal aliquots, and stored frozen.

### Pharmacokinetic assay to measure total human IgG

To measure the human antibody in SD rat or NHP serum samples, a half area black plate (Corning 3694, Corning, NY) was coated with 2 μg/ml of anti-human Fc antibody (Amgen Inc., Thousand Oaks, CA) in PBS and then incubated 12–24 hours at 4°C. The plate was then washed and blocked with I-Block (Life Technologies, Carlsbad, CA) overnight at 4°C. The standards and quality controls (QC) were prepared in rat or NHP serum, and experimental samples were diluted in naïve rat or NHP serum if dilution was required. The standards, QCs, and samples were then diluted 1:20 in a buffer containing PBS, 1M NaCl, 0.5% Tween 20 and 1% bovine serum albumin buffer (5% final rat or NHP serum was used in the assay). The plate was washed three times with approximately 200 μl of 1X KPL buffer (KPL, Gaithersburg, MD), and subsequently 50-μl samples of diluted standards, QCs, and samples were transferred into the anti-human Fc antibody coated plate and incubated for 1.5 hours at room temperature (approximately 25°C). The plate was then washed three times with approximately 200 μl of 1X KPL wash buffer, followed by 50 μl of 100 ng/ml of an orthogonal anti-human Fc antibody horse radish peroxidase (HRP) conjugate (Amgen Inc., Thousand Oaks, CA) in I-Block containing 5% BSA was added and incubated for 1.5 hours at room temperature (approximately 25°C). The plate was washed six times with approximately 200 μl of 1X KPL wash buffer, followed by addition of 50 μl Pico substrate (Thermo Fisher, Rockford, IL), and the chemiluminescent signal was measured using a SpectraMax (Molecular Devices, Sunnyvale, CA) plate reader. Serum concentration data were analyzed using non-compartmental methods with WinNonLin (Enterprise version 5.1.1, 2006, Pharsight Corp. Mountain View, CA).

### Immunogenecity assay to measure the ADA

ADAs to the human antibodies were measured in NHP serum and Sprague Dawley rat serum samples using the Universal Indirect Species-Specific Assay (UNISA) as previously described [22]. Briefly, the 96-well standard binding plate (MSD, Gaithersburg,MD, USA) was coated overnight with 1 μg/ml human IgG in PBS (35 μL/well). The positive controls consisted of 100 and 500 ng/ml of NHP or rat anti-human IgG Fc chimeric antibody spiked into pooled normal NHP or rat serum, respectively. The assay positive controls and serum samples were diluted 1:200 in an assay buffer (5× milk diluent/block (Kirkegaard and Perry Laboratories “KPL,”Gaithersburg, MD, USA)). The coated and blocked plates (blocked with assay buffer, 200 μl/well overnight) were washed on day two with 1× wash buffer (KPL, Gaithersburg, MD, USA) and then the diluted assay controls and serum sample were added (100 μl/well) to the plate and incubated for approximately 3 hours. Plates were washed, and ruthenylated mouse anti-NHP IgG Fc or rabbit anti-rat IgG Fc antibody was added (0.5 μg/ml, 35 μl/well) and incubated for approximately 30 minutes. Following another wash, 2× T read buffer (MSD, Gaithersburg, MD, USA) was added (150 μl/well). The plates were read using the SECTOR Imager 6000 Instrument (MSD, Gaithersburg, MD, USA) and analyzed utilizing Discovery Workbench software (v2. 0 7.3). The resulting ECL was measured and reported in ECL units. The ECL response of the sample over the ECL response of the background of the assay was captured as signal to noise (S/N). The NHP assay has a sensitivity of 5.8 ng/ml, and the Sprague Dawley rat assay has a sensitivity of 64 ng/ml based on a species specific positive control antibody diluted in neat, negative control NHP or Sprague Dawley rat sera.

### Surface equilibrium FcRn binding assay

Assays were conducted on a Biacore 8K at 25°C. The soluble domains of human, NHP, and rat FcRn molecules were covalently immobilized to the active flow cells of channel 1, 2, and 3, respectively on a CM5 chip via standard amine coupling procedures. The immobilization levels were 1400 Ru, 1700 Ru, and 260 Ru for hFcRn, cFcRn, and rFcRn, respectively. To serve as the blank surface for background subtraction, reference flow cell of each channel went through the same procedures of amine coupling without any protein immobilization. The antibody molecules were serial diluted 5-fold from 3.3 μM to 5 nM in 1xPBS, 0.005% Polysorbate 20, pH 5.5 before passing over both reference and active flow cells in each channel for 2 minutes at a flow rate of 30 μl/min. The binding signals at the end of each injection (association phase) were applied in calculation of Kd using 1:1 steady state affinity model provided in Biacore 8 Evaluation Software (v1.1.1). FcRn surfaces were regenerated every cycle by 1xPBS, pH 7.4 to remove the captured antibodies and measure the dissociation rate.

### Statistical analysis

Rat SC and IV ADAs were analyzed with a standard two-by-two contingency table using Barnard’s exact test. Analysis of variance (ANOVA) was carried out by fitting a regression model using rat PK parameters as the response variable and antibody as a nominal independent variable. SC and IV data were analyzed separately, and measurements were collected from 48 different animals. Dunn-Sidak correction was applied to post hoc tests and tests were limited to the antibody exhibiting the best PK property against all other antibodies with the same isotype. NHP PK parameters were calculated using 17 different animals with measurements repeated at the 1 and 5 mg/kg dose levels. ANOVA was carried out using a mixed effects regression model with rat PK parameters as the response variable, antibody as a nominal independent variable, and animal as a random factor. The 1 and 5 mg/kg dose groups were analyzed separately and post hoc analysis was identical to that described above. Least squares means (LSMeans) were determined for each antibody and differences in estimated LSMeans were reported.

Analysis of descriptive mean ratios was carried out by first log transforming the data and then calculating the log of the ratio. Mean errors were propagated in quadrature with subsequent estimation of the 95% confidence interval. Log ratios and corresponding 95% confidence intervals were back transformed for visual display. A ratio of means was considered different from 1 if the 95% confidence interval did not contain 1. Confidence intervals were Bonferroni corrected to the number of comparisons within the isotype. Pearson's product moment correlation was used to estimate the strength of association between variables.

All *p* values correspond to a null hypothesis of no difference and were considered significant at the 0.05 level. All tests were two-sided. Analyses were performed using Matlab version 9.4.0.813654 (R2018a), The MathWorks, Inc., Natick, Massachusetts, United States.

## Results

### Antibody characterization

Four human immunoglobulins raised against three different antigens, with unrelated complementarity determining regions that do not bind mammalian targets were cloned into both IgG1 and IgG2 frameworks and stably expressed in CHO cells. All eight antibodies were basic with calculated isoelectric points between 8.44 and 9.18, and the final yield after two-column purification was comparable for most antibodies ranging from 0.6 to 1.0 g/l conditioned media (Table 1). Exceptions were IgG1 and IgG2 versions of antibody D which produced relatively low yields of 0.2 and 0.1 g/l respectively, and antibody A in the IgG2 format that yielded 2.0 g/l. All purified antibodies were monomeric with very low aggregate levels, >99% main peak by SE-HPLC, with the exception of the IgG1 version of antibody D, which was 94.2% main peak.

**Table 1:** Summary of human IgG1 and IgG2 antibody characteristics. Characteristics of the human IgG1 (A1-D1) and IgG2 (A2-D2) antibodies used in this study including variable light chain (VL) framework type, variable heavy chain (VH) framework type, antigen (Ag) for the antibodies, as well as the calculated isoelectric point (pI). The production yield after a two-column purification process is listed in grams of antibody per liter of conditioned media, and the final product percent monomer, main peak (MP) purity, was determined by size exclusion high performance chromatography (SEC). DNP is dinitrophenyl and KLH is Keyhole Limpet Hemocyanin.

Ab	Iso-type	VL Type	VH Type	Ag	pI	Yield (g/l)	SEC (%MP)
**A1**	**IgG1**	**VK3** **A27**	**VH4** **4–30.4**	**None**	**8.83**	**0.8**	**99.8**
**B1**	**IgG1**	**VK1** **A30**	**VH4** **4–31**	**DNP**	**8.89**	**0.6**	**99.7**
**C1**	**IgG1**	**VK1** **L5**	**VH3** **3–33**	**DNP**	**9.18**	**0.7**	**99.9**
**D1**	**IgG1**	**VK1** **A30**	**VH1** **1–02**	**KLH**	**9.18**	**0.2**	**94.2**
**A2**	**IgG2**	**VK3** **A27**	**VH4** **4–30.4**	**None**	**8.44**	**2.0**	**99.2**
**B2**	**IgG2**	**VK1** **A30**	**VH4** **4–31**	**DNP**	**8.64**	**0.9**	**99.6**
**C2**	**IgG2**	**VK1** **L5**	**VH3** **3–33**	**DNP**	**9.01**	**1.0**	**99.7**
**D2**	**IgG2**	**VK1** **A30**	**VH1** **1–02**	**KLH**	**9.01**	**0.1**	**99.5**

### Pharmacokinetics in rodents

**Fig 1:**
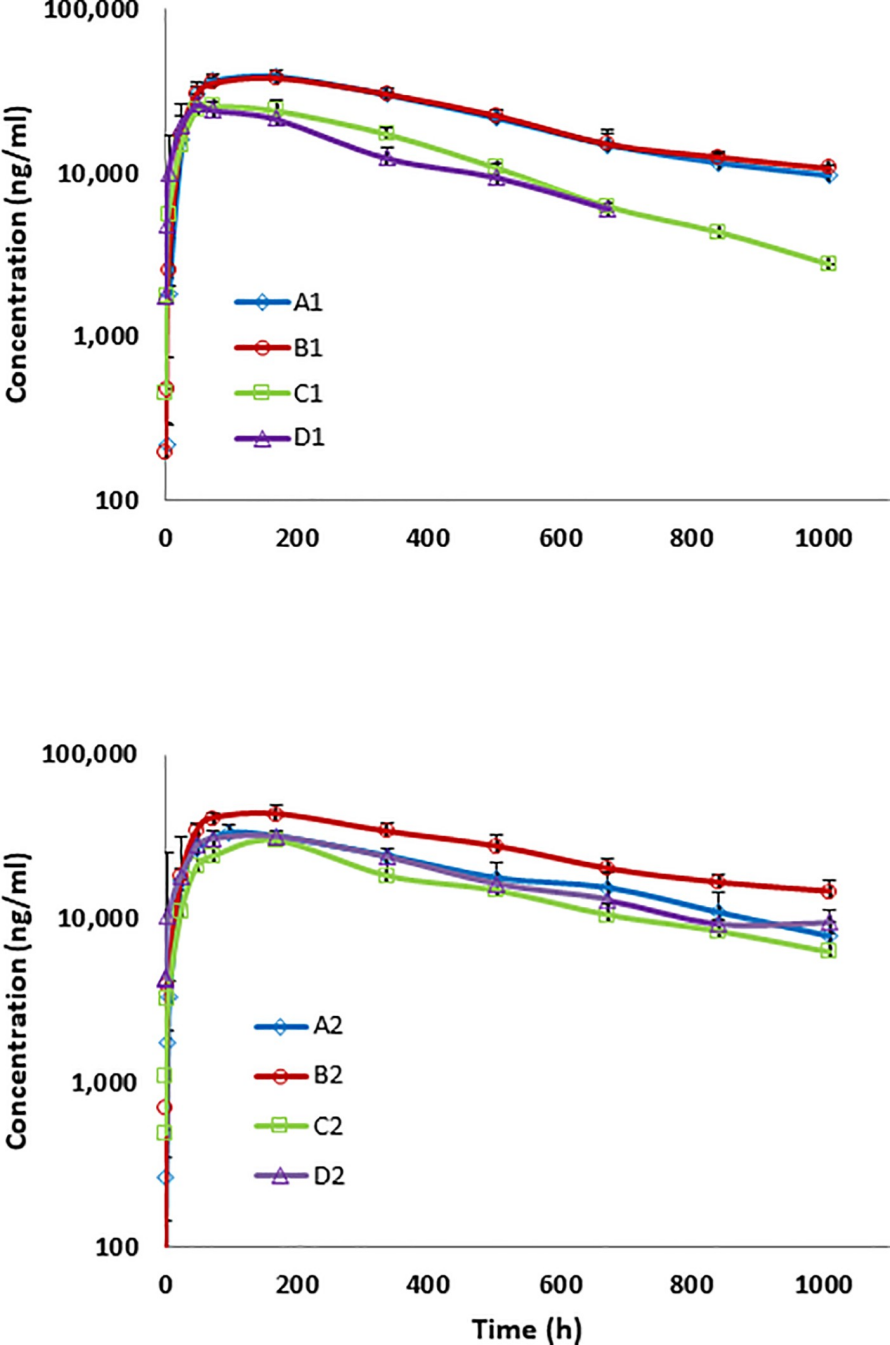
Averaged pharmacokinetic profiles of human IgG1 and IgG2 antibodies in rats. Serum concentrations of human IgG1 (A1-D1) and IgG2 (A2-D2) antibodies over time were determined after subcutaneous administration to Sprague-Dawley rats at 5 mg/kg as measured by a sandwich ELISA over a 42 day period. Each test article was assessed in three animals, which are plotted as the average with error bars representing the standard error of the mean.

All eight antibodies were administered intravenously and subcutaneously to rats (three animals per test article) at 5 mg/kg and serum levels of the antibodies were monitored over 42 days (S1 Fig). Antibodies A and B in both IgG1 (A1, B1) and IgG2 (A2, B2) formats displayed very good pharmacokinetic profiles, and they had better exposures than antibodies C and D with matched isotypes. Likely anti-drug antibody responses (ADA) were observed after two weeks in three animals in groups C1, D1, and D2 (one in each group), and one rat in group D1 was euthanized at day 22 due to an unrelated health issue. As depicted in S1 Fig, the likely ADA response, as defined by an accelerated clearance at two weeks, coincides with a sudden decrease in drug serum concentration after two weeks or more, which was not observed in other subjects in the same cohort. Overall, 11 out of 24 animals had an ADA response over the course of the PK study. The observed excursions from the expected PK profiles are consistent in timing and appearance with typical anti-drug antibody (ADA) responses that induce rapid clearance of the targeted drug [23]. Graphing the average serum level of the IgG1 antibodies for all animals in each group, A1 and B1 display similar PK profiles that are better to those of C1 and D1, which also display profiles similar to each other (Fig 1). For the IgG2 antibodies, the PK profiles were comparable to the IgG1, particularly, A1 and B1 antibodies, with observed attenuated divergence of C2 and D2 compared to C1 and D1.

The PK profile of these eight antibodies were also obtained by intravenous administration in rats at 5 mg/kg. (S2 and S3 Figs). The antibodies A1 and B1 had numerically higher exposures than C1 and D1 similar to the ones observed in SC PK data. The antibodies A2 and B2, had slightly better exposures than C2 and D2 with PK profiles comparable to SC PK data. As expected, the likely ADA response in the IV group was substantially less (4 out of 24 rats) than the SC groups (*Barnard’s exact test*, *n* = 48, *p* = 0.032). Overall, both D1 and D2 were more immunogenic in both IV and SC cohorts than any other antibody. The overall bioavailability for all antibodies were in the rage of 0.9–1.3 (except D2 which was 0.7), and the average bioavailability of all G1 was 1.1 and for G2 was 0.9 which indicate that the absorption kinetics among all 8 antibodies were almost complete and comparable.

The overall difference between the IV and SC clearances and exposures among all isotypes are less than 50%. The exposures of B2, C1, and D2 had the highest variations among others, although, there was no statistically significant. However, there was a statistically significant different between B1 and B2 clearances in IV and SC routs (S3 Fig).

In rats, mean MRT difference was found in the SC route (*F*(7,14) = 3.47, *p* = 0.023) and post hoc analysis revealed antibody B1 was elevated by 279 h compared to D1. No statistical differences were found between the IgG2 antibodies. For the IV route mean MRT differences were also detected (*F*(7,14) = 7.22, *p* < 0.001) with a mean difference of 139 and 118 hours between antibodies A1 –D1 and A2 –C2, respectively.

Differences in mean clearance were observed in both the SC and IV routes (*F*(7,13) = 15.3, *p* < 0.001, and *F*(7,14) = 49.3, *p* < 0.001, respectively). The mean clearance of C1 and D1 was elevated by 0.19 and 0.31 mL/h/kg compared to B1, and C2 was elevated by 0.15 mL/h/kg compared to B2 in the SC cohort. IV administration resulted in elevated mean clearance between IgG1 antibodies B1 –C1 (0.29 mL/h/kg) and B1 –D1 (0.31 mL/h/kg)) along with elevated levels found between the IgG2 antibody pair B2 –C2 (0.15 mL/h/kg).

Mean exposures were different in both the SC and IV routes (*F*(7,15) = 7.80, *p* < 0.001, and *F*(7,15) = 25.3, *p* < 0.001, respectively). The mean clearance of IgG1 antibody A1 was elevated relative to C1 and D1 by 10.3 and 13.8 mg •h/kg, respectively in the SC group. Similarly, increased mean exposure was detected for B2 relative to C2 and D2 by 13.4 and 15.6 mg •h/kg, respectively. In the IV group mean clearance levels were elevated between IgG1 antibody B1 and antibodies C1 (0.29 mL/h/kg) and D1 (0.31 mL/h/kg)), along with elevated levels between the IgG2 antibody B2 and antibodies C2 (0.15 mg •h/kg) and D2 (0.15 mg •h/kg).

**Fig 2:**
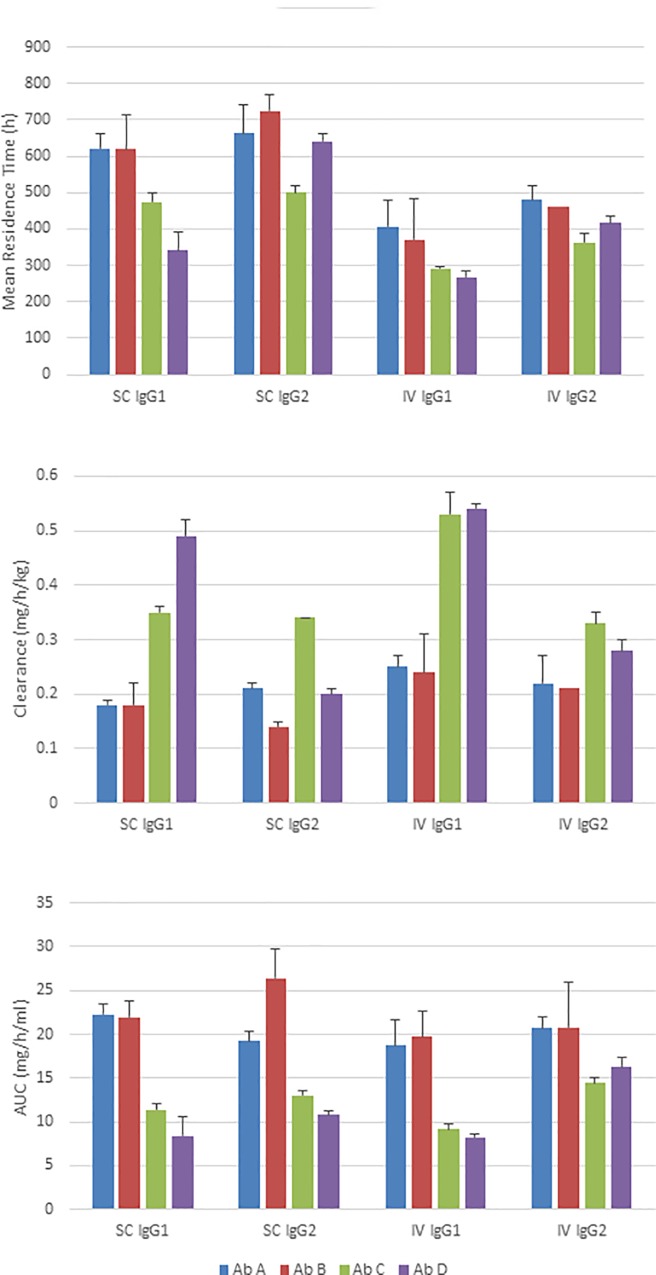
Pharmacokinetics of human IgG1 and IgG2 antibodies in rats. Pharmacokinetic parameters were determined for the IgG1 (A1-D1) and IgG2 (A2-D2) antibody panels after subcutaneous (SC) administration to Sprague-Dawley rats at 5 mg/kg (n = 3). The mean *in vivo* residence time (MRT), clearance rate (CL/F) and area under the curve from the time of the first antibody serum concentration measurement to the last measurement (AUC_0-t_) were calculated for each animal individually by non-compartmental analysis (NCA) and then averaged showing the standard error of the mean (antibodies A1 and A2 blue bars, B1 and B2 red bars, C1 and C2 green bars, D1 and D2 purple bars). For the subcutaneous antibody D2 (MRT) and (CL/F) were calculated using only one animal due to the likely ADA response in the other two animals.

Overall, IgG1 antibody half-lives ranged from 211 to 402 hours with clearance rates of 0.18 to 0.53 ml/h/kg, and all calculated PK parameters for A1 and B1 were numerically better to those of C1 and D1 (Fig 2). Similar to the IgG1 antibodies, the IgG2 antibodies, A2 and B2 generally showed better PK attributes compared to C2 and D2. The average PK parameter values for all four IgG1 antibodies were comparable to the average of all four IgG2 antibodies, differing by no more than 25% (S1 Table); however, IgG1 antibodies C1 and D1 have numerically higher clearance rates than their IgG2 counterparts C2 and D2 after subcutaneous injection (Fig 2).

**Fig 3:**
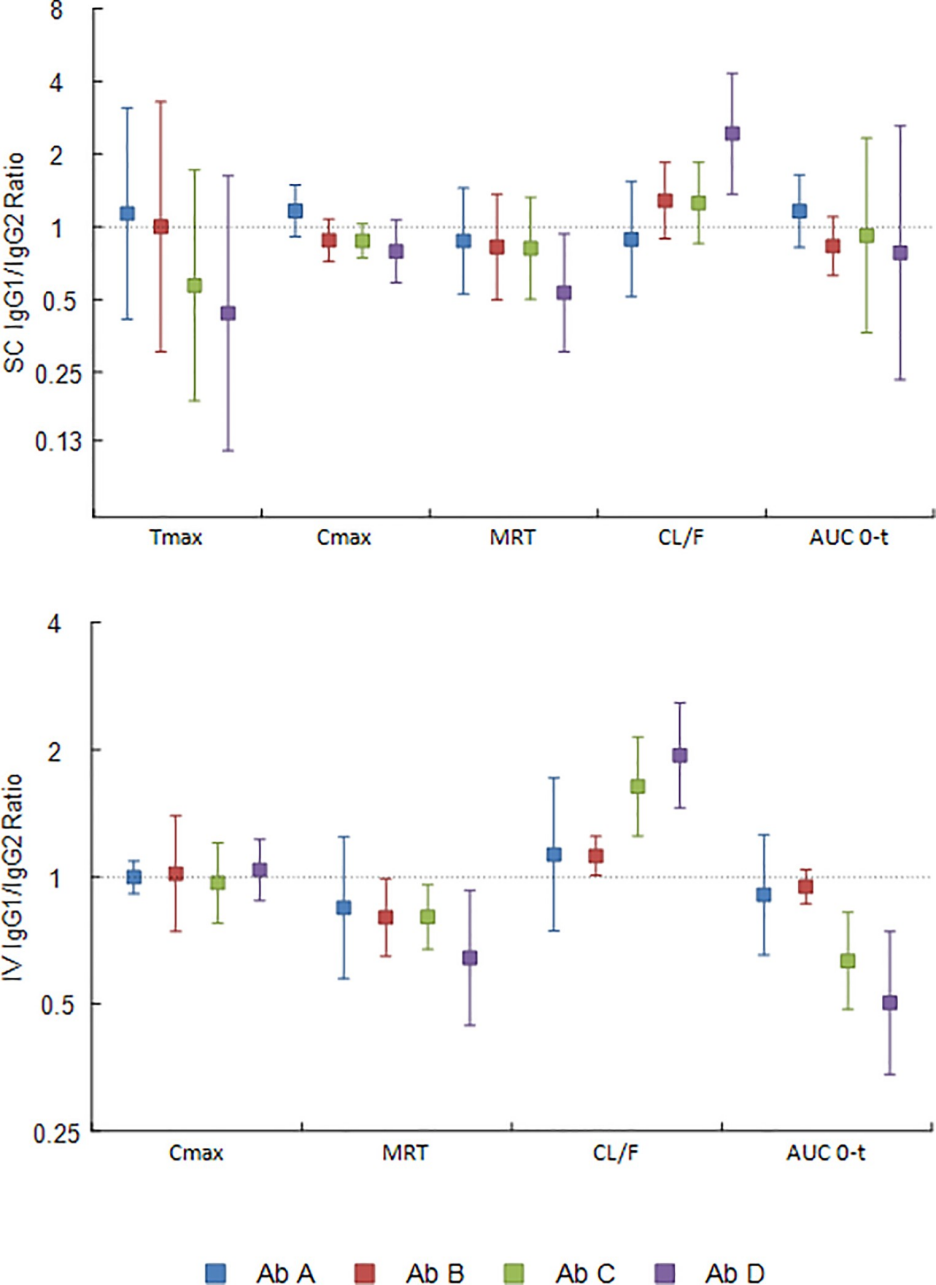
Ratio of human IgG1 to IgG2 antibody pharmacokinetic parameters from rats. The ratio of the pharmacokinetic parameters of human IgG1 to the human IgG2 antibodies with matched complementary determining regions after subcutaneous (SC) administration to Sprague-Dawley rats at 5 mg/kg was calculated using the data from S1 Table (antibodies A1 and A2 blue bars, B1 and B2 red bars, C1 and C2 green bars, D1 and D2 purple bars). A ratio of means was considered different from 1 if the 95% confidence interval did not contain 1.

The ratios of the PK parameters of IgG1 antibodies to their IgG2 counterparts were not different from 1 for A, B and C after SC dosing; however, antibody D ratios were different from one for MRT and clearance. Following IV dosing, IgG1/IgG2 ratios were not different from 1 for A and B (except for B MRT ratio). In contrast C and D ratios were all different from 1 with the exception of Cmax (Fig 3).

### Pharmacokinetics in non-human primates

All eight antibodies were administered subcutaneously to NHPs (two animals per test article, except A2 which had three animals) first at 1 mg/kg, then after a 56 day washout phase, the animals received a subsequent injection at 5 mg/kg. Serum levels of the antibodies were monitored for a total of 112 days. All antibodies displayed normal PK profiles after both injections, and no prominent excursions indicative of overt ADA were observed (S4 Fig). Although the B1, C2 and D2 groups showed some divergence in serum levels between the animals after the 1 mg/kg administration, the difference was not observed in the same animals after the 5 mg/kg injection. Subsequent ADA analysis of the serum samples at days 56 and 112 for all animals were ADA negative (S2 Table).

**Fig 4:**
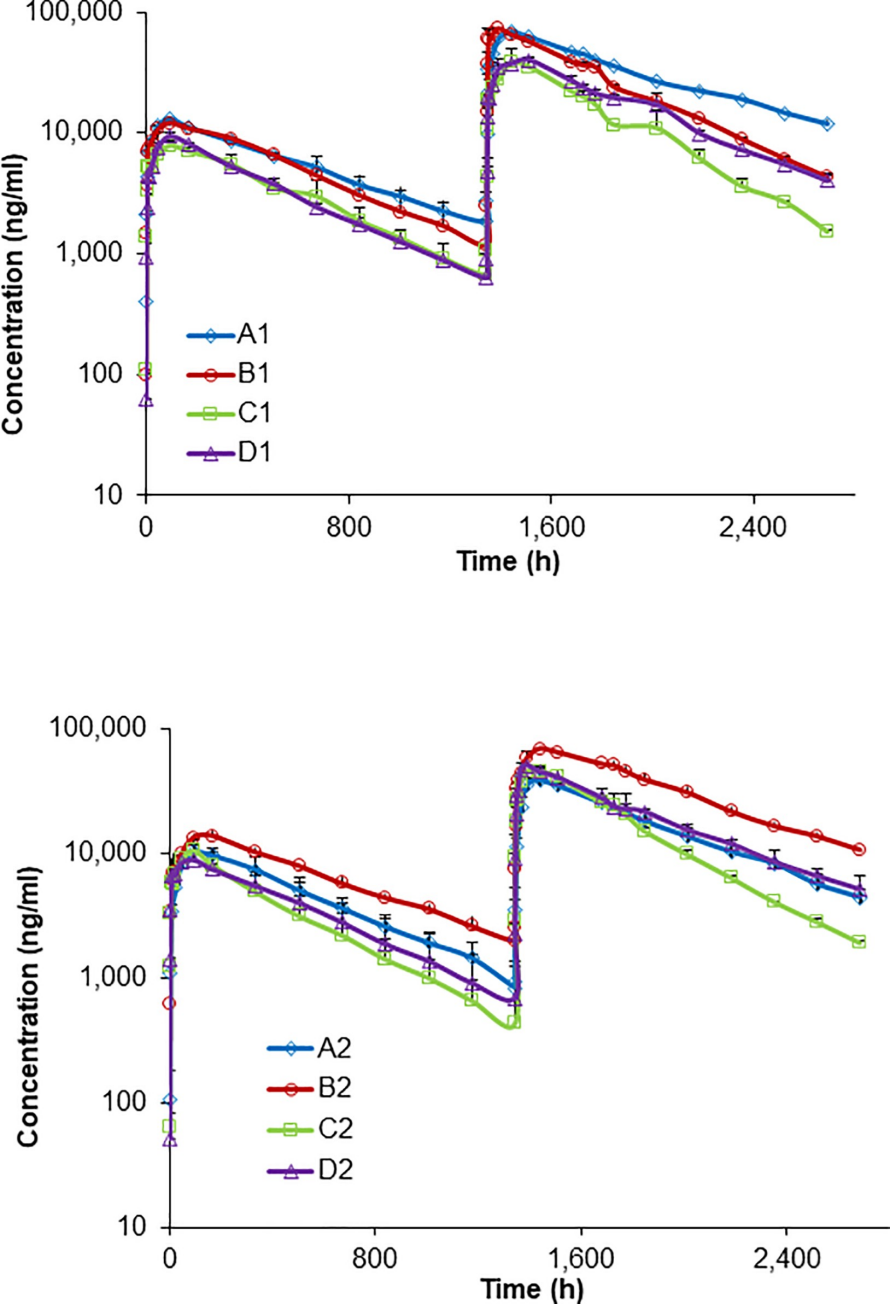
Averaged pharmacokinetic profiles of human IgG1 and IgG2 antibodies in NHPs. Serum concentrations of human IgG1 (A1-D1) and IgG2 (A2-D2) antibodies were determined over time after subcutaneous administration to NHPs first at 1 mg/kg then at 5 mg/kg 56 days later. Each test article was assessed in two animals, which are plotted as the average with error bars representing the standard error of the mean.

For the IgG1 antibodies, A1 and B1 had better exposures compared to C1 and D1 at both 1 mg/kg and the subsequent 5 mg/kg administrations; however, B1 had about 20% lower exposure at 5 mg/kg as compared to A1 (Fig 4). For the IgG2 antibodies, B2 had the best exposure and lowest clearance as compared to A2, C2 and D2 at both 1 mg/kg and the subsequent 5 mg/kg dose, with C2 consistently tracking at the bottom of the group. However, similar to antibody B1, C2 showed some divergence in PK 18 days after the 5 mg/kg injection.

**Fig 5:**
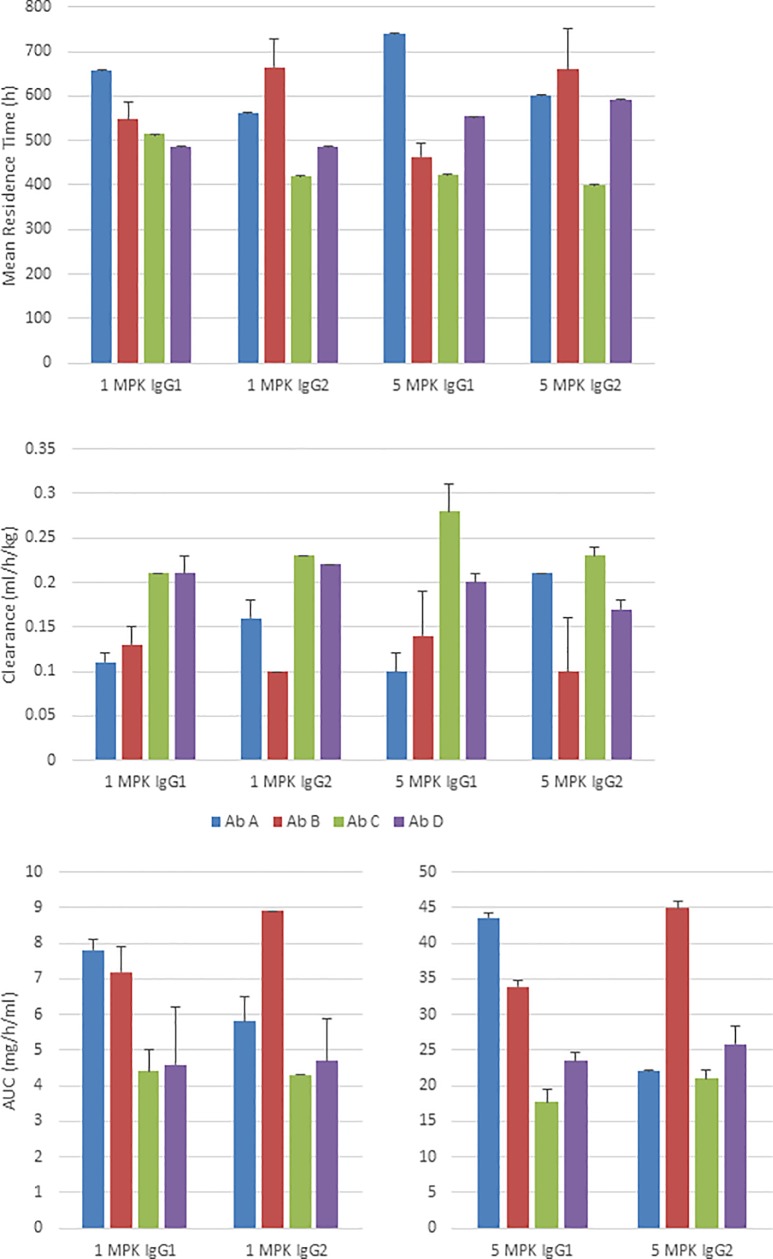
Pharmacokinetics of human IgG1 and IgG2 antibodies in NHPs. Pharmacokinetic parameters were determined for the IgG1 (A1-D1) and IgG2 (A2-D2) antibody panels after subcutaneous administration to NHPs first at 1 mg/kg (n = 2 for all groups except A2 with n = 3), then at 5 mg/kg 56 days later. Each test article was assessed in two animals. The mean *in vivo* residence time (MRT), clearance rate (CL/F) and area under the curve from the time of the first antibody serum concentration measurement to the last measurement (AUC_0-t_) were calculated for each animal individually using non-compartmental methods and then averaged (antibodies A1 and A2 blue bars, B1 and B2 red bars, C1 and C2 green bars, D1 and D2 purple bars). Data were calculated separately for the 1 mg/kg and 5 mg/kg administrations. At 5 mg/kg the half-lives of the IgG1 antibodies ranged from 265 to 502 hours and the clearance rates ranged from 0.10 to 0.28 ml/h/kg, which were closely matched to the values observed at 1 mg/kg. Also consistent with the 1 mg/kg dose, at 5 mg/kg antibodies A1 and B1 PK attributes were numerically better to C1 and D1, particularly for PK parameters such as clearance rates and exposure (Fig 5). Antibody A1’s exposure was significantly elevated (*F*(7, 9) = 7.13, *p* = 0.004) with mean difference of 3.37 and 3.25 mg •h/mL relative to antibodies C1 and D1 respectively at the 1 mg/kg dose, while at the 5 mg/kg dose antibody A1’s exposure was superior (*F*(7, 9) = 47.9, *p* < 0.001) compared to antibodies B1, C1, and D2 with mean differences of 9.65, 25.9, and 20.1 mg •h/mL, respectively. Clearance was not statistically different between the IgG1 antibodies at the 1 mg/kg dose, but antibody A1’s clearance was significantly lowered (*F*(7, 9) = 7.69, *p* = 0.003) at the 5 mg/kg dose with decreased mean differences of 0.18 and 0.10 mL/h/kg compared to C1 and D1, respectively.

The half-lives of the IgG1 antibodies in NHPs at 1 mg/kg ranged from 322 to 440 hours, with clearance rates ranging from 0.11 to 0.21 ml/h/kg (Fig 5, S3 Table). Antibodies A1 and B1 showed comparable PK parameter values, which were generally better to those of C1 and D1, particularly with regard to clearance rate and exposure. For the IgG2 antibodies at 1 mg/kg, the half-lives ranged from 292 to 435 hours and the clearance rates ranged from 0.10 to 0.23 ml/h/kg. Antibody B2 numerically had the best PK attributes compared to the other antibodies, and antibody A2 PK parameter values were between B2 and the C2 and D2 antibodies. Comparing the average PK parameters of the IgG1 and IgG2 antibodies at 1 mg/kg in NHPs, the differences, excluding T_max_, were all less than 9% (S3 Table).

At 5 mg/kg, the IgG2 antibodies displayed half-lives of 264 to 434 hours with clearance rates of 0.10 to 0.23 ml/h/kg, again very comparable to the 1 mg/kg dose. Also consistent with the 1 mg/kg dose, the PK parameters of B2 were substantially better than those of A2, C2 and D2 at 5 mg/kg, particularly with regard to clearance and exposure. Antibody B2 demonstrated significantly better exposure ((*F*(7, 9) = 47.9, *p* < 0.001) with mean differences of 3.04, 4.55, and 4.21 mg •h/mL corresponding to B2, C2 and D3 at the 1 mg/kg dose, and 22.9, 23.9, and 19.0 mg •h/mL at the 5 mg/kg dose, respectively) and the lowest clearance (*F*(7, 9) = 15.5, *p* < 0.001) as compared to A2, C2 and D2 with decreased mean differences compared to antibodies A2, C2, and D2 of 0.11, 0.13 and 0.08 mL/h/kg respectively for the 5 mg/kg dose. No significant differences in clearance was found for the IgG2 antibodies at the 1 mg/kg dose.

**Fig 6:**
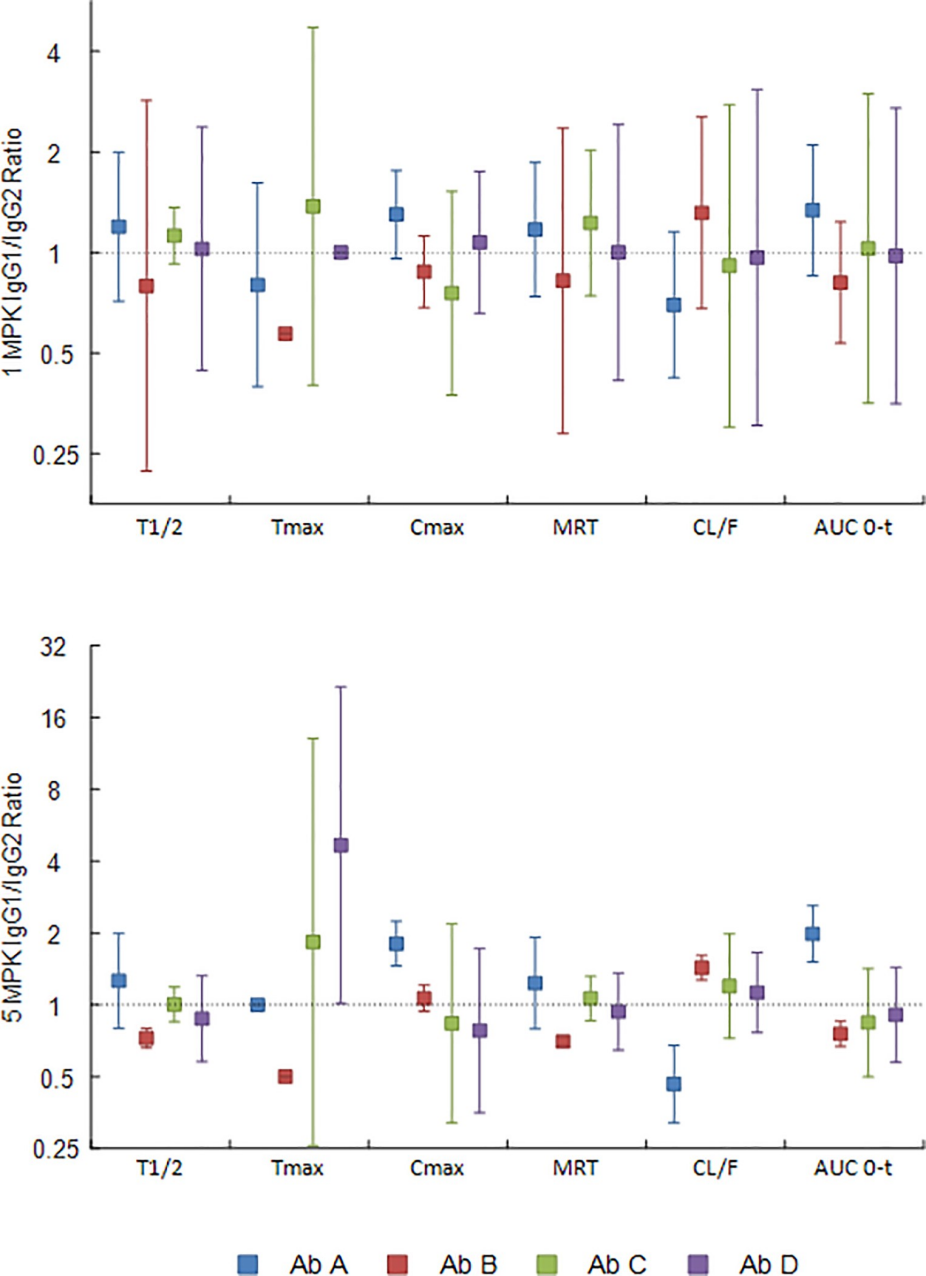
Ratio of human IgG1 to IgG2 antibody pharmacokinetic parameters from NHPs. The ratio of the pharmacokinetic parameters of human IgG1 to the human IgG2 antibodies with matched complementarity determining regions after subcutaneous administration to NHPs first at 1 mg/kg, then at 5 mg/kg 56 days later, were calculated separately for the 1 mg/mg and 5 mg/kg doses using the data from S3 Table (antibodies A1 and A2 blue bars, B1 and B2 red bars, C1 and C2 green bars, D1 and D2 purple bars). A ratio of means was considered different from 1 if the 95% confidence interval did not contain 1.

The average PK parameters of the IgG1 and IgG2 antibodies at 5 mg/kg were very similar, with the exception of T_max_, differing by no more than 8% (S3 Table). The ratios of the PK parameters of IgG1 to IgG2 were not different from 1 across all antibody pairs except for the Tmax B ratio in the 1 mg/kg cohort. For the 5 mg/kg dose group no differences from 1 were found for antibodies C and D; however, antibody B ratios were all different from 1 except for Cmax. For antibody A, ratios were different from 1 for Cmax, clearance, and exposure (Fig 6).

**Fig 7:**
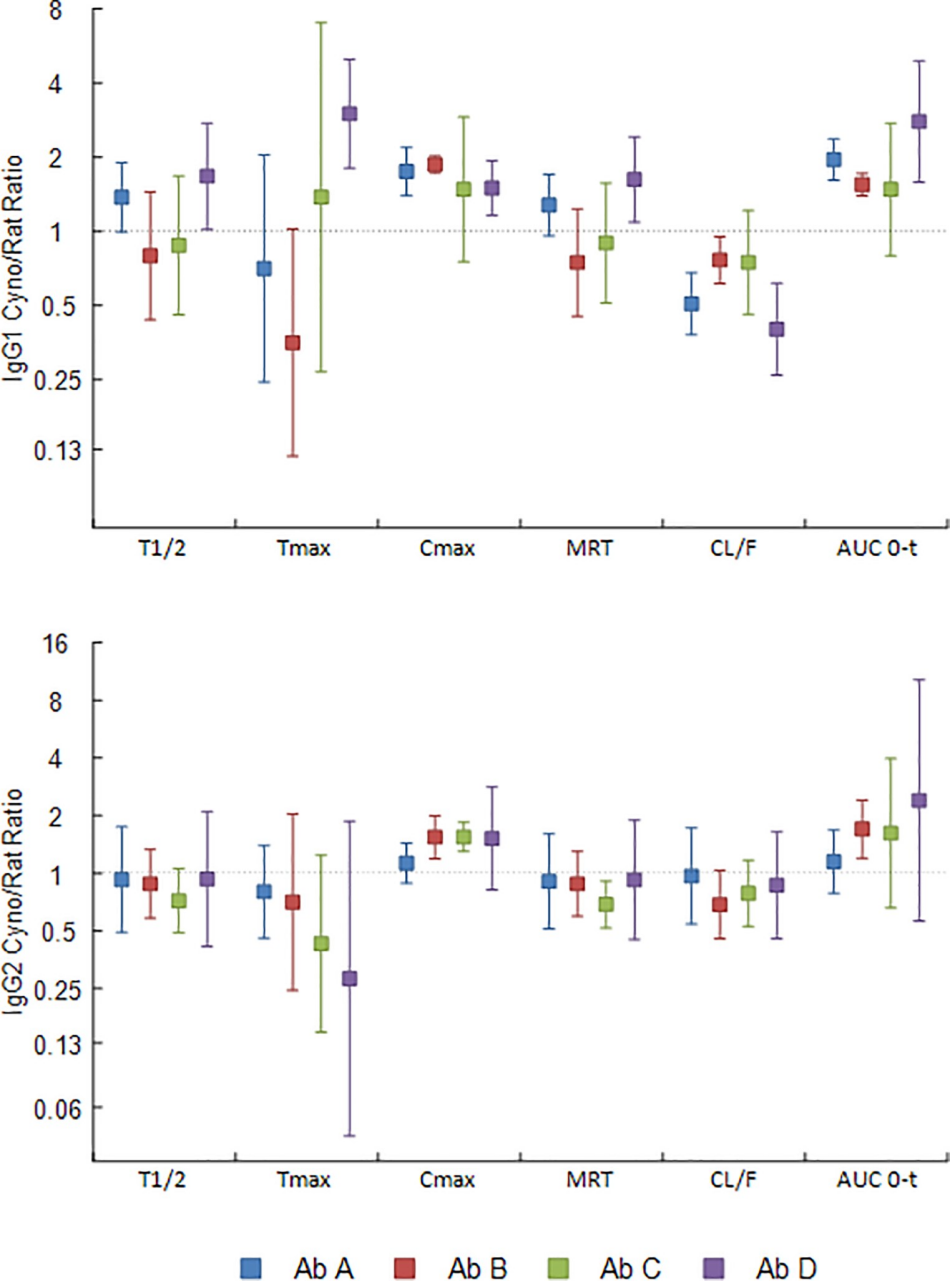
Comparison of rat and NHP pharmacokinetic parameters for human IgG1 and IgG2 antibodies. The ratio of the pharmacokinetic parameters comparing NHPs to rats after subcutaneous administration of human antibodies at 5 mg/mg were calculated for the IgG1 (A1-D1) and IgG2 (A2-D2) antibodies separately using the data from S1 and S3 Tables (antibodies A1 and A2 blue bars, B1 and B2 red bars, C1 and C2 green bars, D1 and D2 purple bars). A ratio of means was considered different from 1 if the 95% confidence interval did not contain 1.

When comparing the PK parameters of NHPs and rats, both at the 5 mg/kg dose, all were within 2-fold of each other (Fig 7) except for isotype B and C Tmax ratios, and isotype D ratios for Tmax, clearance and AUC. It should be noted that the apparent clearance (CL/F) was used for NHPs to calculate the interspecies clearance ratio. Similar NHP to rat PK parameters ratios are observed using both NHP and rat PK parameters and apparent clearances from SC dosing.

**Fig 8:**
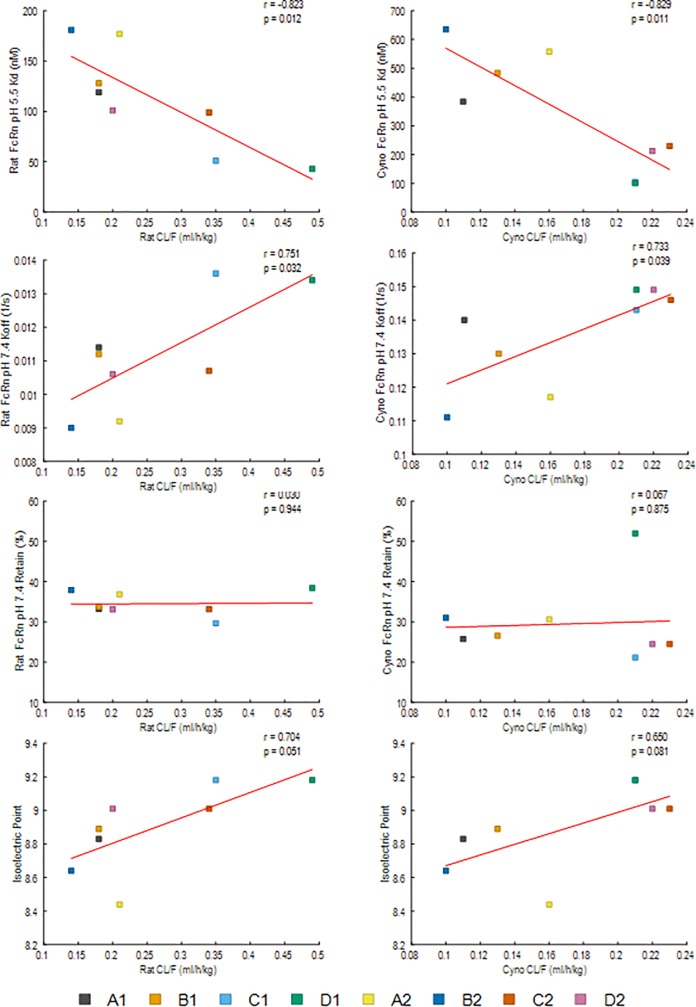
Comparison of plasma clearance rates and FcRn binding and dissociation properties. FcRn binding characteristics of the human IgG1 (A1-D1) and IgG2 (A2-D2) antibodies used in this study were determined by surface plasmon resonance using soluble human FcRn, NHP FcRn and rat FcRn at pH 5.5. Dissociation was carried out at pH 7.4. FcRn molecules were covalently attached to the surface then the antibody molecules were passed over both reference and active flow cells. The binding properties were compared to the clearance rates in NHPs and rats as previously described.

Binding of the antibodies to the extracellular domain of human, NHP and rat FcRn was determined by surface plasmon resonance. All IgG1 antibodies consistently bound tighter to all species of FcRn at pH 5.5 than their IgG2 counterparts with the IgG2 Kd’s being 31% to 135% higher (average 84%) (S6 and S7 Figs). Antibodies with higher affinities to FcRn at pH 5.5 also tended to have higher rates of clearance in both rats and NHPs with strong and significant correlations found between Kd and clearance rate as well as Koff and clearance rate (top 4 panels, Fig 8). In addition, antibodies with faster initial off rates at pH 7.4 tended to have slower clearance rates *in vivo*. However, no association was found between the fraction of antibody remaining bound to the SPR surface after the initial release phase and in vivo clearance rates (Fig 8). An increasing numerical relationship between isoelectric point and clearance rates *in vivo* was observed but the association was not significant.

## Discussion

Comparison of the PK profile of a panel of four unrelated non-binding antibodies as isotype matched pairs, IgG1 and IgG2, in both rats and NHPs, allowed for examination of the impact of antibody isotype and model species on PK without the complication of antigen interactions *in vivo*. Although none of the antibodies examined in this study bind mammalian antigens, there were significant differences between the clearance rates of the antibodies within the same isotype group (up to 2.7-fold in rats and up to 2.8-fold in NHPs). Since these antibodies possess matched isotype frameworks, and showed similar thermal stability (S4 Table), the primary differences are the CDRs, which are therefore likely responsible for the PK differences independent of antigen binding. It is possible that some CDR sequences are more or less susceptible to chemical or proteolytic degradation *in vivo* than others, and/or there may be differential uptake by cellular degradation pathways. It has also been reported the structure of CDRs and the charge patches may impact the FcRn binding and therefore may impact the PK [44, 45, 46, 47, 48]. We did observe a significant correlation between FcRn affinity at pH 5.5 and clearance rates; however, it was inverse of what might be expected with higher affinity antibodies clearing faster. In addition, the initial dissociation rate at pH 7.4 unexpectedly showed a significant correlation between fast dissociation and faster clearance rates [49, 50, 51]. Hence, the higher FcRn affinity at pH = 7.4 may be preventing the antibodies from dissociating after reaching the cell surface protecting them from circulation-based clearance mechanisms, ultimately leading to lower clearance rates. This indicates that it is more important to engineer slower FcRn off rates at pH 7.4 than higher affinity at pH 5.5 to obtain improved pharmacokinetic properties, and the CDRs significantly influence the FcRn interactions. The numerically increasing relationship between higher calculated antibody isoelectric points and faster clearance rates, also indicates a potential path for CDR engineering that could be undertaken.

Since the pattern of PK profiles observed for the four different antibodies was maintained between rodents and non-human primates, the antibody clearance mechanisms are likely conserved between these species. In the absence of target mediated clearance, maintaining similar antibody constant regions may further enable the engineering of CDR mediated PK improvements. Therefore, it may be possible to directly translate improved rodent PK to improved performance of therapeutic candidates in humans without the need for non-human primates in the early molecule screening studies [52, 53].

As predicted by allometric scaling [14, 54, 55], the antibodies examined in this study show higher C_max_ (1.5-fold), higher AUC (1.8-fold or 2 fold obtained from IV dose) and lower apparent clearance (1.4-fold or 1.8 fold of rat CL obtained from IV dose) values in NHPs compared to rats, which is in the expected range based on allometric scaling (C_max_ and AUC is expected to be approximately two times higher in NHP as compared to rat based on body surface area [41]. Although the C_max_, AUC and clearance inter-species ratios were relatively consistent among the antibodies, the T_max_ values and inter-species ratios showed substantial variability depending on the antibody. In addition, the T_max_ also displayed variability between different isotypes of the same antibody, and this was observed in both rodents and non-human primates. This variability is likely due to difference in the absorption rates of antibodies from the subcutaneous space.

Antibody isotype had minimal impact on antibody PK performance, since, with the notable exception of the more variable T_max_, the ratio of IgG1 to IgG2 PK parameter values are near one for the majority of the cases. Furthermore, this observation was consistent in both rats as well as NHPs. Antibody D displayed the highest level of deviation from PK parameter ratios of one, and this antibody was consistently the most problematic in the panel yielding the lowest production levels (3 to 20-fold lower), the most immunogenic in rats, and the lowest to second lowest PK performance in all groups and species tested. This may indicate that antibody D has an inherent instability related to its CDR sequences, which negatively impacts the PK performance. The clearance and AUC ratio of G1/G2 for antibody A at 5 mg/kg in non-human primates was about 2; however, it should be noted that the G2 version of antibody A was done in separate study and two of animals in that study developed likely ADA once they were dosed at 5 mg/kg, which may explain why the AUC ratio for the antibody A was much higher than the other three antibodies.

The PK attributes of these eight antibodies in the rat IV and SC were comparable with clearances and exposures among all isotypes are less than 50%. Although the exposures of B2, C1, and D2 had the highest variations among others, the differences weren’t statistically significant; however, B1 and B2 clearances between the IV and SC were statistically different (S5 Fig). Since, there was no significant ADA response in either route of administration for the B1 and B2 groups, and the difference between IV and SC groups could be attributed to the variations among animals and PK studies. In addition, there were high level of ADA response for C and D groups, and this could be an additional factor in the overall variabilities observed in IV and SC PK attributes.

It has been reported that high pI is associated with poor PK in antibodies [9], and consistent with this finding the better performing pair of antibodies (A and B) have lower pIs than the poorer performing counterparts (C and D). However, it should be noted that all four antibodies have favorable PK properties compared to those in the literature. The antibodies presented here have half-lives in NHPs ranging from 264 to 502 hours (average 362 hours) and clearance rates from 0.10 to 0.28 ml/h/kg (average 0.18 ml/h/kg); which exceed the half-lives for many antibodies in monkeys (29 to 299 hours, average 156 hours) and clearances from 0.07 to 1.14 ml/h/kg (average 0.69 ml/h/kg) [2, 3, 4, 5, 6, 7, 8]. These antibodies even fall within the range of antibodies engineered for enhanced PK, which have half-lives ranging from 113 to 746 hours (average 495 hours) [5, 6, 7, 28, 29]. The exceptional production level and stability of antibodies A, B and C ensure favorable molecular attributes that may contributed to their excellent PK performance, and while pI likely has a role in antibody PK, it is not necessarily a dominant factor.

Although target mediated clearance of the antibodies was not a factor in this study, likely ADA responses had an impact on the later phase of the PK profiles in some animals. This deviation was more overt in rats, with as a sudden drop in antibody serum levels occurring 14 days post-injection in a subset of animals, which is consistent with the timeframe expected for a typical ADA response [23] and this was confirmed by the ADA analysis. The ADA responses to antibodies C and D in rats was against the CDR sequences, while antibodies A or B showed significantly lower ADA responses, which were primarily against the constant regions. In contrast, the ADA response was much less common in the NHP PK study resulting in less impact on exposure, which may be due to the higher level of similarity between human and NHP antibodies resulting in a higher level of immune-tolerance. In general, this panel of antibodies, particularly A, B and C, may have unusually low immunogenicity, since immune responses to human antibodies in NHPs is common, and the immune response in rats was less frequent than typically observed (the frequency of the ADA response in this work as well as many other internal observations). It is possible that antigenicity is related to antibody stability, since antibodies A, B & C were selected for their exceptional production levels and stability during processing and in formulation, while antibody D, the most immunogenic, was not, and it scored the lowest of this group in regard to these properties. In addition, the lack of antigen binding *in vivo*, may also contribute to a lower immunogenicity.

## Conclusions

This study demonstrated that the PK of antibodies is dependent on the content of the CDRs, even in the absence of antigen binding, and the CDR dependent properties are predictable between rodents and primates. These observations open the possibility that CDRs could be engineered for enhanced PK, which could be assessed in rodents with a good probability the improvements will translate well when the antibodies are employed in humans. This enables minimizing the need for NHP PK studies to screen engineered therapeutic candidates, reserving the use of NHPs for the final lead candidates. Furthermore, these antibodies demonstrated that there is little difference in the PK of human IgG1 and IgG2 antibodies; therefore, the isotype providing the desired level of effector function (high or low respectively) can be chosen to address the therapeutic need without concern for the impact on PK.

## Supporting information

S1 Fig Pharmacokinetic profiles of human IgG1 and IgG2 antibodies in individual rats. Serum concentrations of human IgG1 (A1-D1) and IgG2 (A2-D2) antibodies were determined over time after subcutaneous administration at 5 mg/kg to Sprague-Dawley rats as measured by a sandwich ELISA over a 42 day period. Each test article was assessed in three animals, which are plotted individually, with a 10 ng/ml lower limit of quantification (LLOQ).
(TIF)

S2 Fig Pharmacokinetic profiles of human IgG1 and IgG2 antibodies in individual rats. Serum concentrations of human IgG1 (A1-D1) and IgG2 (A2-D2) antibodies were determined over time after intravenous administration at 5 mg/kg to Sprague-Dawley rats as measured by a sandwich ELISA over a 42 day period. Each test article was assessed in three animals, which are plotted individually, with a 10 ng/ml lower limit of quantification (LLOQ).
(TIF)

S3 Fig Averaged pharmacokinetic profiles of human IgG1 and IgG2 antibodies in rats. Serum concentrations of human IgG1 (A1-D1) and IgG2 (A2-D2) antibodies over time were determined after intravenous administration to Sprague-Dawley rats at 5 mg/kg as measured by a sandwich ELISA over a 42 day period. Each test article was assessed in three animals, which are plotted as the average with error bars representing the standard error of the mean.
(TIF)

S4 Fig Pharmacokinetic profiles of human IgG1 and IgG2 antibodies in individual NHPs. Serum concentrations of human IgG1 (A1-D1) and IgG2 (A2-D2) antibodies were determined over time after subcutaneous administration to NHPs first at 1 mg/kg followed by a second administration at 5 mg/kg 56 days later. Antibody level measurements were measured over the 102 day study by sandwich ELISA. Each test article was assessed in two animals, except A2 with three, which are plotted individually, with an LLOQ of 10 ng/ml.
(TIF)

S5 Fig Comparison of pharmacokinetic profiles of human IgG1 and IgG2 antibodies in rats obtained from IV and SC administration. The ratio of the clearances and exposures obtained in rats after intravenous and subcutaneous administration of human antibodies at 5 mg/mg were calculated for the IgG1 (A1-D1) and IgG2 (A2-D2) antibodies separately using the data from S1 and S3 Tables.
(TIF)

S6 Fig Equilibrium FcRn binding and dissociation by surface plasmon resonance. The FcRn binding characteristics of the human IgG1 (A1-D1) and IgG2 (A2-D2) antibodies used in this study were determined by surface plasmon resonance using soluble human FcRn, NHP FcRn and rat FcRn at pH 5.5. Dissociation was carried out at pH 7.4. FcRn molecules were covalently attached to the surface then the antibody molecules were passed over both reference and active flow cells.
(TIF)

S7 Fig FcRn Dissociation by surface plasmon resonance. The FcRn binding characteristics of the human IgG1 (A1-D1) and IgG2 (A2-D2) antibodies used in this study were determined by surface plasmon resonance using soluble human FcRn, NHP FcRn and rat FcRn binding at pH 5.5. Dissociation was carried out at pH 7.4 and the rate calculation was done over the range of 121–160 seconds for human and NHP FcRn and between 124–430 seconds for rat FcRn. FcRn molecules were covalently attached to the surface then the antibody molecules were passed over both reference and active flow cells.
(TIF)

S1 Table Pharmacokinetics of human IgG1 and IgG2 antibodies in rats. Pharmacokinetic parameters were determined for the IgG1 (A1-D1) and IgG2 (A2-D2) antibody panels after subcutaneous administration (A) or intravenous administration (B) to Sprague-Dawley rats at 5 mg/kg (n = 3). The *in vivo* terminal half-life (T_1/2_), time for maximum serum levels (T_max_), maximum serum concentration achieved (C_max_), mean *in vivo* residence time (MRT), clearance rate (CL/F) and area under the curve from the time of the first antibody serum concentration measurement to the last measurement (AUC_0-t_) were calculated for each animal individually by non-compartmental analysis (NCA) and then averaged. Listed errors are the standard error of the mean. *For the subcutaneous antibody D2, (T_1/2_), (MRT) and (CL/F) were calculated using only one animal due to the likely ADA response in the other two animals. **For the intravenous antibody D2 and intravenous antibody C1, (T_1/2_), (MRT) and (CL/F) were calculated using only two animals due to the likely ADA response in the other animal.
(DOCX)

S2 Table Anti-Drug antibody levels against human IgG1 and IgG2 antibodies in Rat and NHP PK samples. The Anti-Drug Antibody (ADA) levels against human IgG1 and IgG2 antibodies in rat and NHP PK study samples were determined by UNISA. The ADA analysis were conducted using the last PK time points (1008 hr post dosed samples following 5 mg/kg administration of the human antibodies in rat and 1344 hr post dosed samples following 1 or 5 mg/kg administration of the human antibodies in NHP). The signal to noise (S/N) responses were calculated using the signal from average negative control samples. *This animal was ADA positive prior to dosing with an S/N of 5.3.
(DOCX)

S3 Table Pharmacokinetics of human IgG1 and IgG2 antibodies in NHPs. Pharmacokinetic parameters were determined for the IgG1 (A1-D1) and IgG2 (A2-D2) antibody panels after subcutaneous administration to NHPs first at 1 mg/kg (n = 2 for all groups except A2 with n = 3), then at 5 mg/kg 56 days later. Each test article was assessed in two animals. The *in vivo* terminal half-life (T_1/2_), time for maximum serum levels (T_max_), maximum serum concentration achieved (C_max_), mean *in vivo* residence time (MRT), clearance rate (CL/F) and area under the curve from the time of the first antibody serum concentration measurement to the last measurement (AUC_0-t_) were calculated for each animal individually using non-compartmental methods and then averaged. Data were calculated separately for the 1 mg/kg and 5 mg/kg administrations.
(DOCX)

S4 Table Differential scanning calorimetry of antibodies. Differential scanning calorimetry (DSC) was performed on a Malvern MicroCal VP-Capillary DSC. See Fig 8. The following parameters were used: scanning range: 10–100°C; scanning rate: 1°C/min; pre-scan thermostat 15 min. Typically 400 μL of 1 mg/mL sample is consumed for each analysis. Data was processed in Origin 7 software.
(DOCX)
