# Supplementary figures and images for: Pharmacokinetic comparison of a diverse panel of non-targeting human antibodies as matched IgG1 and IgG2 isotypes in rodents and non-human primates
Source: PLoS One. 2019 May 23;14(5):e0217061. doi: 10.1371/journal.pone.0217061 (PMC6533040; doi:10.1371/journal.pone.0217061)

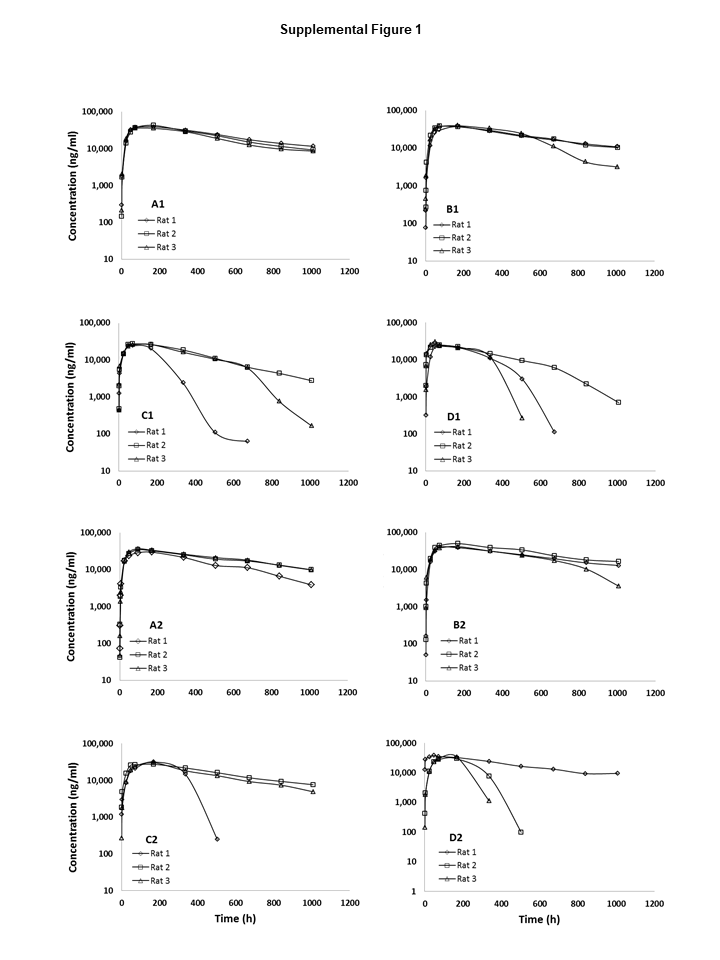

Supplement: S1 Fig — Serum concentrations of human IgG1 (A1-D1) and IgG2 (A2-D2) antibodies were determined over time after subcutaneous administration at 5 mg/kg to Sprague-Dawley rats as measured by a sandwich ELISA over a 42 day period. Each test article was assessed in three animals, which are plotted individually, with a 10 ng/ml lower limit of quantification (LLOQ). (TIF) [file pone.0217061.s001.TIF]

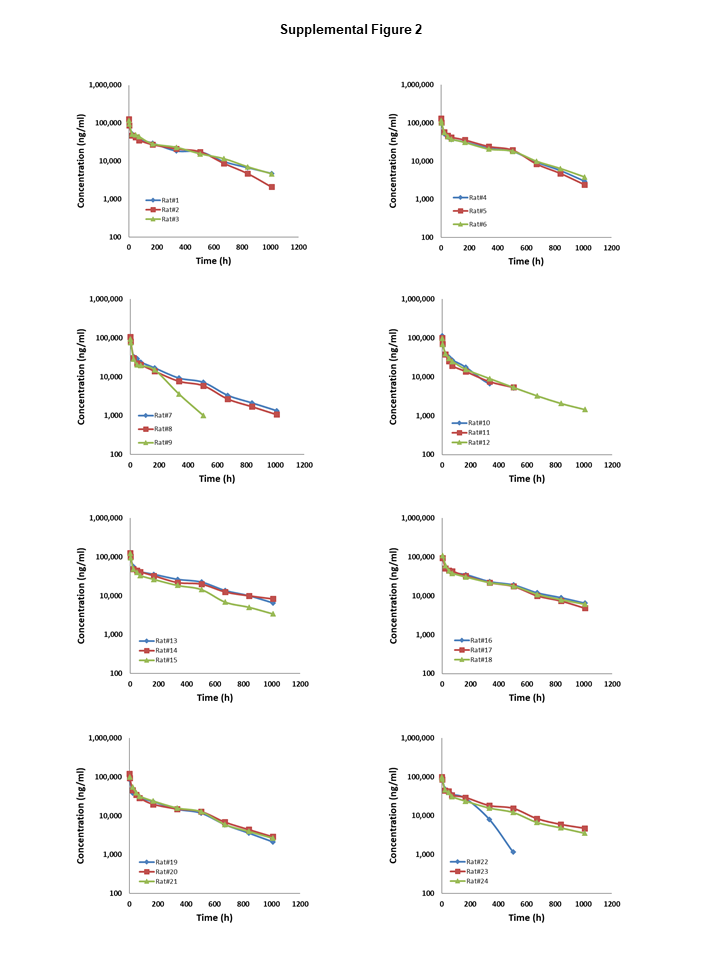

Supplement: S2 Fig — Serum concentrations of human IgG1 (A1-D1) and IgG2 (A2-D2) antibodies were determined over time after intravenous administration at 5 mg/kg to Sprague-Dawley rats as measured by a sandwich ELISA over a 42 day period. Each test article was assessed in three animals, which are plotted individually, with a 10 ng/ml lower limit of quantification (LLOQ). (TIF) [file pone.0217061.s002.TIF]

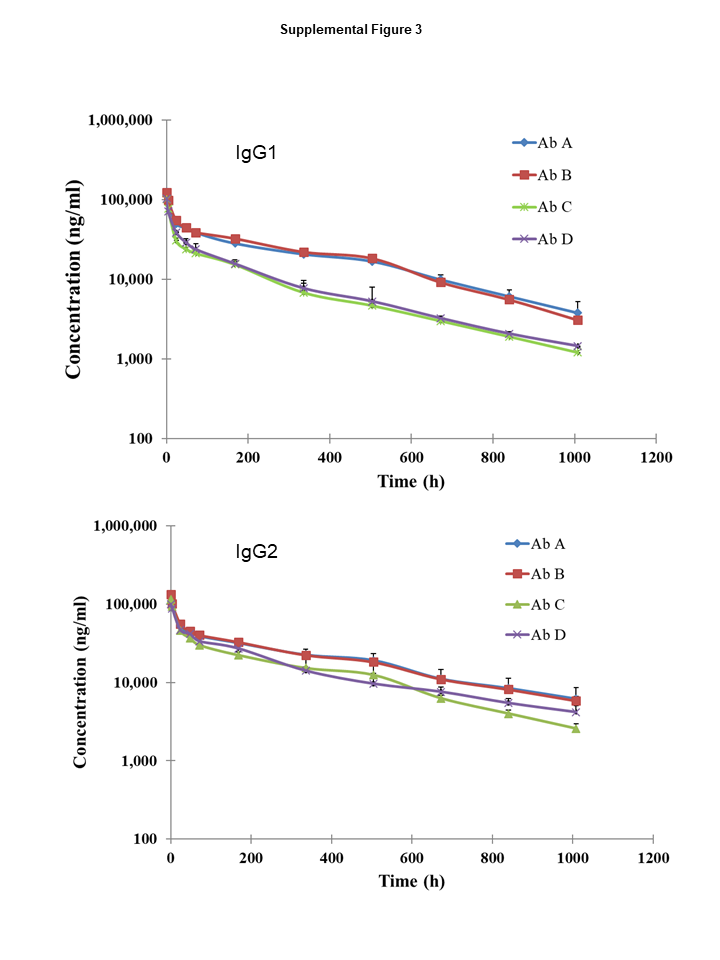

Supplement: S3 Fig — Serum concentrations of human IgG1 (A1-D1) and IgG2 (A2-D2) antibodies over time were determined after intravenous administration to Sprague-Dawley rats at 5 mg/kg as measured by a sandwich ELISA over a 42 day period. Each test article was assessed in three animals, which are plotted as the average with error bars representing the standard error of the mean. (TIF) [file pone.0217061.s003.TIF]

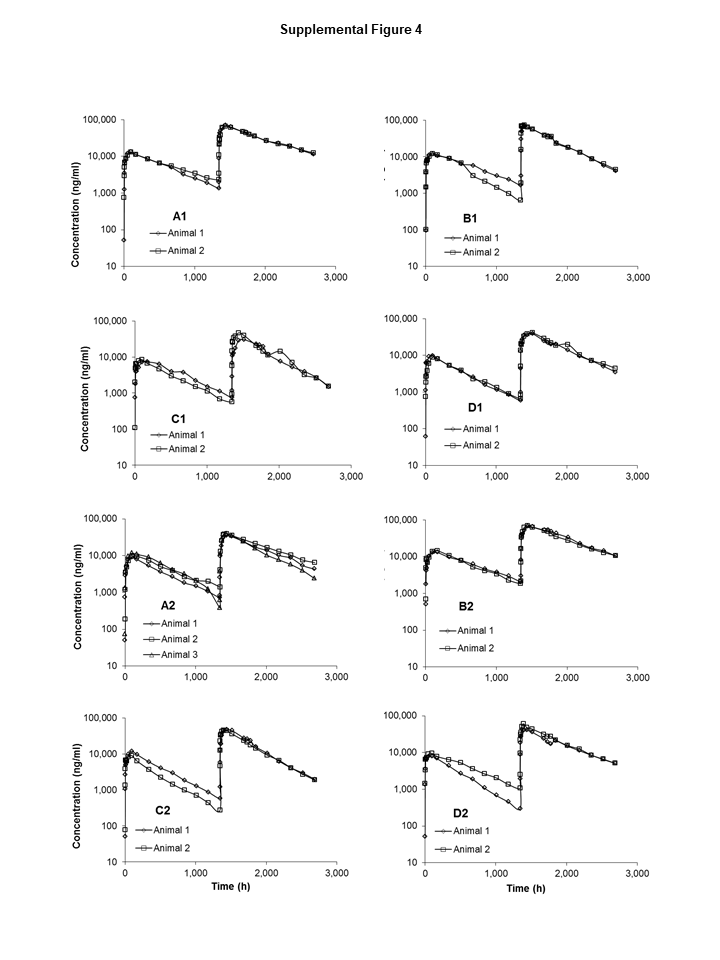

Supplement: S4 Fig — Serum concentrations of human IgG1 (A1-D1) and IgG2 (A2-D2) antibodies were determined over time after subcutaneous administration to NHPs first at 1 mg/kg followed by a second administration at 5 mg/kg 56 days later. Antibody level measurements were measured over the 102 day study by sandwich ELISA. Each test article was assessed in two animals, except A2 with three, which are plotted individually, with an LLOQ of 10 ng/ml. (TIF) [file pone.0217061.s004.TIF]

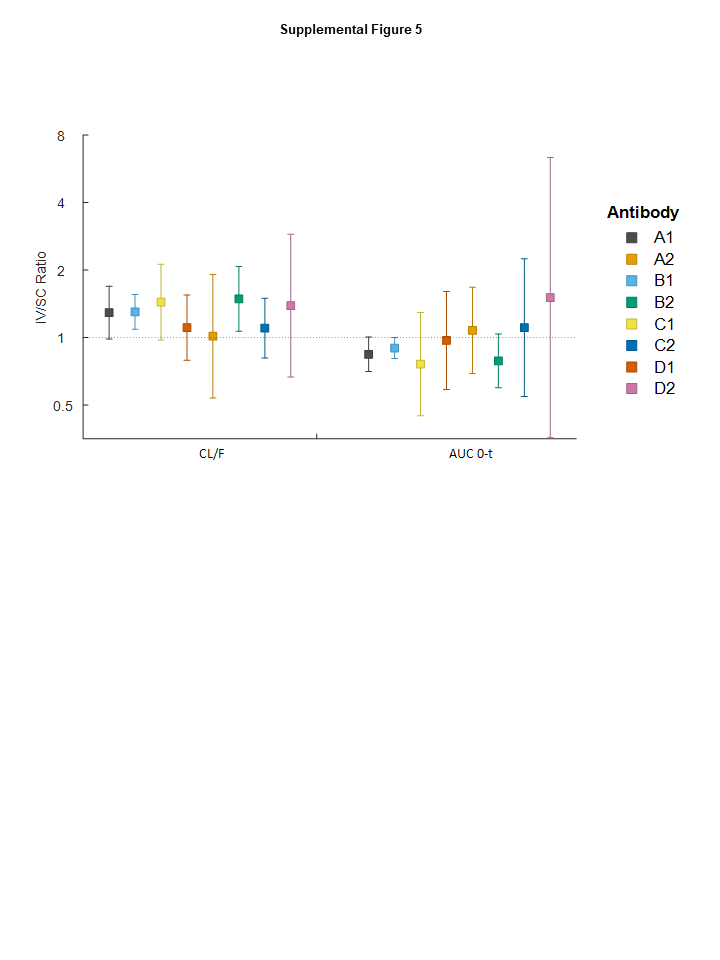

Supplement: S5 Fig — The ratio of the clearances and exposures obtained in rats after intravenous and subcutaneous administration of human antibodies at 5 mg/mg were calculated for the IgG1 (A1-D1) and IgG2 (A2-D2) antibodies separately using the data from S1 and S3 Tables. (TIF) [file pone.0217061.s005.TIF]

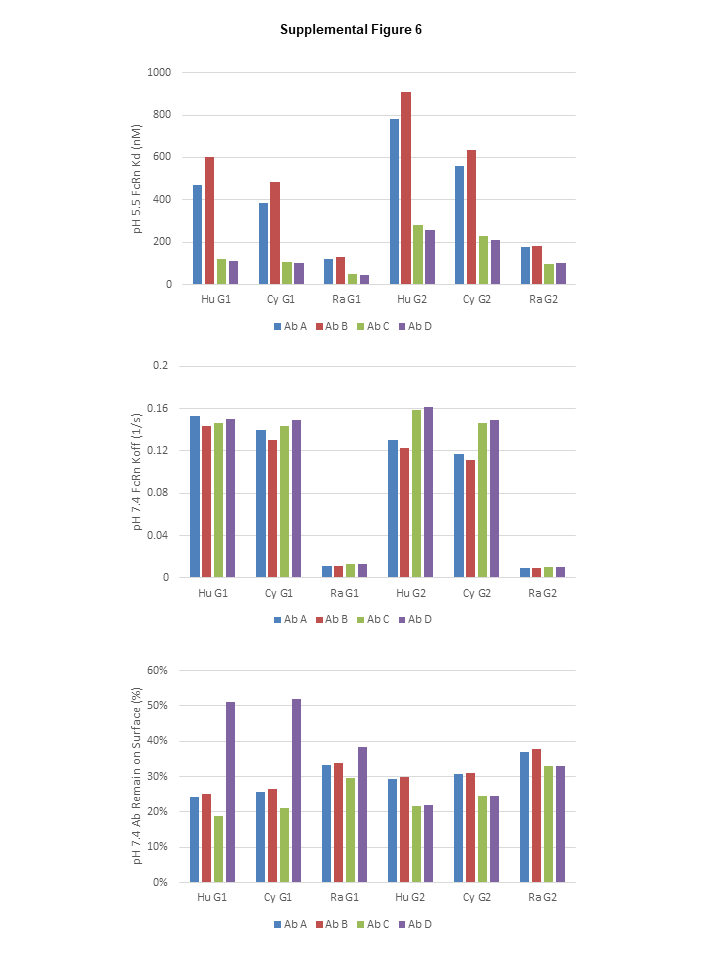

Supplement: S6 Fig — The FcRn binding characteristics of the human IgG1 (A1-D1) and IgG2 (A2-D2) antibodies used in this study were determined by surface plasmon resonance using soluble human FcRn, NHP FcRn and rat FcRn at pH 5.5. Dissociation was carried out at pH 7.4. FcRn molecules were covalently attached to the surface then the antibody molecules were passed over both reference and active flow cells. (TIF) [file pone.0217061.s006.TIF]

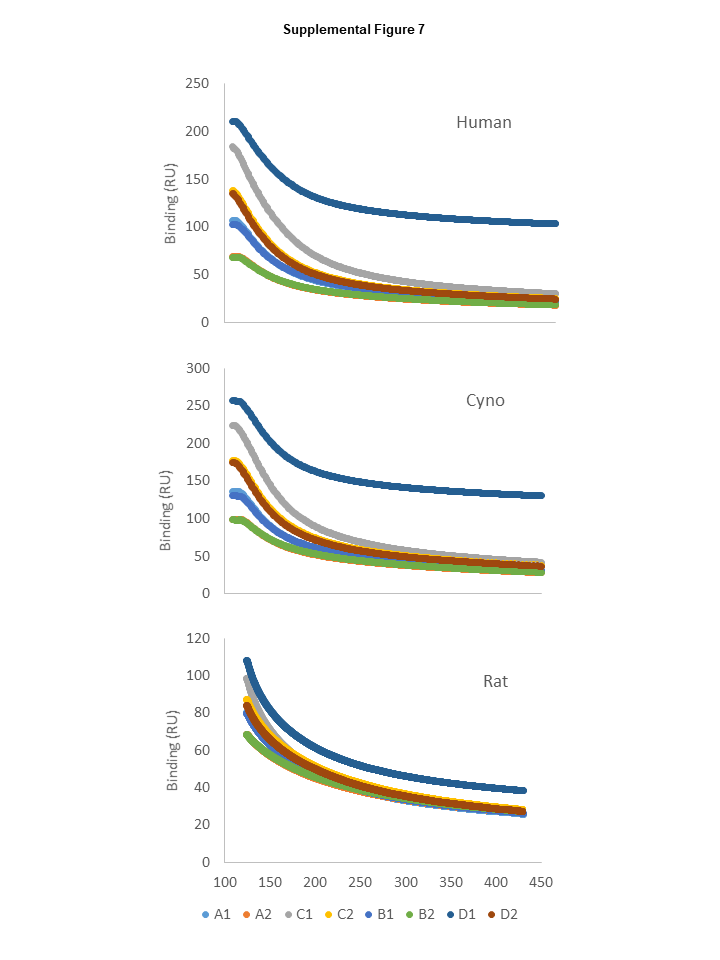

Supplement: S7 Fig — The FcRn binding characteristics of the human IgG1 (A1-D1) and IgG2 (A2-D2) antibodies used in this study were determined by surface plasmon resonance using soluble human FcRn, NHP FcRn and rat FcRn binding at pH 5.5. Dissociation was carried out at pH 7.4 and the rate calculation was done over the range of 121–160 seconds for human and NHP FcRn and between 124–430 seconds for rat FcRn. FcRn molecules were covalently attached to the surface then the antibody molecules were passed over both reference and active flow cells. (TIF) [file pone.0217061.s007.TIF]
