# Supplementary material for: Pharmacokinetic comparison of a diverse panel of non-targeting human antibodies as matched IgG1 and IgG2 isotypes in rodents and non-human primates
Source: PLoS One. 2019 May 23;14(5):e0217061. doi: 10.1371/journal.pone.0217061 (PMC6533040; doi:10.1371/journal.pone.0217061)
Supplement: S1 Table — Pharmacokinetic parameters were determined for the IgG1 (A1-D1) and IgG2 (A2-D2) antibody panels after subcutaneous administration (A) or intravenous administration (B) to Sprague-Dawley rats at 5 mg/kg (n = 3). The in vivo terminal half-life (T1/2), time for maximum serum levels (Tmax), maximum serum concentration achieved (Cmax), mean in vivo residence time (MRT), clearance rate (CL/F) and area under the curve from the time of the first antibody serum concentration measurement to the last measurement (AUC0-t) were calculated for each animal individually by non-compartmental analysis (NCA) and then averaged. Listed errors are the standard error of the mean. *For the subcutaneous antibody D2, (T1/2), (MRT) and (CL/F) were calculated using only one animal due to the likely ADA response in the other two animals. **For the intravenous antibody D2 and intravenous antibody C1, (T1/2), (MRT) and (CL/F) were calculated using only two animals due to the likely ADA response in the other animal. (DOCX) [file pone.0217061.s008.docx]

**S1 Table A**

| **Ab** | **T_1/2_**  **(h)** | **T_max_**  **(h)** | **C_max_**  **(μg/ml)** | **MRT**  **(h)** | **CL/F**  **(ml/h/kg)** | **AUC_0-t_**  **(mg·h/ml)** |
| --- | --- | --- | --- | --- | --- | --- |
| **A1** | **365 ± 32** | **136 ± 32** | **39.0 ± 2.0** | **580 ± 44** | **0.19 ± 0.01** | **22.3 ± 1.1** |
| **B1** | **395 ± 57** | **136 ± 32** | **38.8 ± 0.6** | **620 ± 77** | **0.18 ± 0.01** | **21.9 ± 0.6** |
| **C1** | **303 ± 56** | **96 ± 37** | **26.1 ± 0.8** | **474 ± 73** | **0.37 ± 0.01** | **11.9 ± 1.1** |
| **D1** | **216 ± 29** | **56 ± 8** | **26.8 ± 1.7** | **341 ± 37** | **0.49 ± 0.05** | **8.4 ± 1.3** |
| **Average** | **320 ± 91** | **106 ± 59** | **32.7 ± 2.8** | **514 ± 120** | **0.31 ± 0.06** | **16.0 ± 2.0** |
|  |  |  |  |  |  |  |
| **A2** | **430 ± 68** | **120 ± 24** | **33.5 ± 2.1** | **664 ± 93** | **0.21 ± 0.04** | **19.2 ± 1.9** |
| **B2** | **493 ± 54** | **136 ± 54** | **44.1 ± 3.0** | **751 ± 78** | **0.14 ± 0.02** | **26.4 ± 2.4** |
| **C2** | **369 ± 34** | **168 ± 0** | **29.8 ± 1.3** | **582 ± 42** | **0.29 ± 0.03** | **13.0 ± 2.7** |
| **D2*** | **444 ± NA** | **128 ± 40** | **34.0 ± 2.6** | **641 ± NA** | **0.20 ± NA** | **10.8 ± 4.4** |
| **Average** | **434 ± 94** | **138 ± 57** | **35.4 ± 4.7** | **660 ± 128** | **0.21 ± 0.05** | **17.3 ± 6.0** |

**S1 Table B**

| **Ab** | **T_1/2_**  **(h)** | **T_max_**  **(h)** | **C_max_**  **(μg/ml)** | **MRT**  **(h)** | **CL/F**  **(ml/h/kg)** | **AUC_0-t_**  **(mg·h/ml)** |
| --- | --- | --- | --- | --- | --- | --- |
| **A1** | **287 ± 24** | **N/A** | **125 ± 3.5** | **406 ± 25** | **0.24 ± 0.01** | **18.7 ± 0.6** |
| **B1** | **259 ± 18** | **N/A** | **123 ± 4.4** | **371 ± 20** | **0.24 ± 0.00** | **19.7 ± 0.4** |
| **C1**** | **223 ± 2** | **N/A** | **106 ± 0.7** | **291 ± 7** | **0.53 ± 0.04** | **9.1 ± 0.7** |
| **D1** | **211 ± 20** | **N/A** | **104 ± 4.7** | **268 ± 23** | **0.54 ± 0.02** | **8.2 ± 0.7** |
| **Average** | **245 ± 36** | **N/A** | **115 ± 7.4** | **334 ± 40** | **0.39 ± 0.05** | **13.9 ± 1.2** |
|  |  |  |  |  |  |  |
| **A2** | **346 ± 36** | **N/A** | **125 ± 1.1** | **481 ± 52** | **0.22 ± 0.03** | **20.7 ± 2.1** |
| **B2** | **336 ± 17** | **N/A** | **121 ± 11.5** | **463 ± 21** | **0.21 ± 0.01** | **20.8 ± 0.5** |
| **C2** | **266 ± 13** | **N/A** | **110 ± 6.4** | **363 ± 16** | **0.32 ± 0.01** | **14.4 ± 0.4** |
| **D2**** | **307 ± 10** | **N/A** | **100 ± 0.3** | **417 ± 18** | **0.28 ± 0.02** | **16.2 ± 1.2** |
| **Average** | **314 ± 43** | **N/A** | **114 ± 13** | **431 ± 61** | **0.26 ± 0.04** | **18.0 ± 2.5** |
