# Supplementary material for: Pharmacokinetic comparison of a diverse panel of non-targeting human antibodies as matched IgG1 and IgG2 isotypes in rodents and non-human primates
Source: PLoS One. 2019 May 23;14(5):e0217061. doi: 10.1371/journal.pone.0217061 (PMC6533040; doi:10.1371/journal.pone.0217061)
Supplement: S2 Table — The Anti-Drug Antibody (ADA) levels against human IgG1 and IgG2 antibodies in rat and NHP PK study samples were determined by UNISA. The ADA analysis were conducted using the last PK time points (1008 hr post dosed samples following 5 mg/kg administration of the human antibodies in rat and 1344 hr post dosed samples following 1 or 5 mg/kg administration of the human antibodies in NHP). The signal to noise (S/N) responses were calculated using the signal from average negative control samples. *This animal was ADA positive prior to dosing with an S/N of 5.3. (DOCX) [file pone.0217061.s009.docx]

| **Ab** | **Animal** | **Dose** | **S/N** | **Finding** | **Specificity** |
| --- | --- | --- | --- | --- | --- |
| **B1** | **Rat 1** | **5 MPK** | **0.3** | **Negative** | **N/A** |
| **B1** | **Rat 2** | **5 MPK** | **0.4** | **Negative** | **N/A** |
| **B1** | **Rat 3** | **5 MPK** | **11** | **Positive** | **CDR & Fc** |
| **C2** | **Rat 1** | **5 MPK** | **494** | **Positive** | **CDR** |
| **C2** | **Rat 2** | **5 MPK** | **0.8** | **Negative** | **N/A** |
| **C2** | **Rat 3** | **5 MPK** | **1.6** | **Negative** | **N/A** |
| **D2** | **Rat 1** | **5 MPK** | **0.4** | **Negative** | **N/A** |
| **D2** | **Rat 2** | **5 MPK** | **575** | **Positive** | **CDR** |
| **D2** | **Rat 3** | **5 MPK** | **589** | **Positive** | **CDR** |
|  |  |  |  |  |  |
| **A1** | **NHP 1** | **1 MPK** | **N/A** | **Negative** | **N/A** |
| **B1** | **NHP 1** | **1 MPK** | **N/A** | **Negative** | **N/A** |
| **C1** | **NHP 1** | **1 MPK** | **N/A** | **Negative** | **N/A** |
| **B2** | **NHP 1** | **1 MPK** | **N/A** | **Negative** | **N/A** |
| **C2** | **NHP 1** | **1 MPK** | **N/A** | **Negative** | **N/A** |
| **D2** | **NHP 1** | **1 MPK** | **5.3** | **Positive*** | **N/A** |
| **A1** | **NHP 1** | **5 MPK** | **N/A** | **Negative** | **N/A** |
| **B1** | **NHP 1** | **5 MPK** | **N/A** | **Negative** | **N/A** |
| **C1** | **NHP 1** | **5 MPK** | **N/A** | **Negative** | **N/A** |
| **B2** | **NHP 1** | **5 MPK** | **N/A** | **Negative** | **N/A** |
| **C2** | **NHP 1** | **5 MPK** | **N/A** | **Negative** | **N/A** |
| **D2** | **NHP 1** | **5 MPK** | **N/A** | **Negative** | **N/A** |
