# Supplementary material for: Pharmacokinetic comparison of a diverse panel of non-targeting human antibodies as matched IgG1 and IgG2 isotypes in rodents and non-human primates
Source: PLoS One. 2019 May 23;14(5):e0217061. doi: 10.1371/journal.pone.0217061 (PMC6533040; doi:10.1371/journal.pone.0217061)
Supplement: S3 Table — Pharmacokinetic parameters were determined for the IgG1 (A1-D1) and IgG2 (A2-D2) antibody panels after subcutaneous administration to NHPs first at 1 mg/kg (n = 2 for all groups except A2 with n = 3), then at 5 mg/kg 56 days later. Each test article was assessed in two animals. The in vivo terminal half-life (T1/2), time for maximum serum levels (Tmax), maximum serum concentration achieved (Cmax), mean in vivo residence time (MRT), clearance rate (CL/F) and area under the curve from the time of the first antibody serum concentration measurement to the last measurement (AUC0-t) were calculated for each animal individually using non-compartmental methods and then averaged. Data were calculated separately for the 1 mg/kg and 5 mg/kg administrations. (DOCX) [file pone.0217061.s010.docx]

| **Ab** | **T_1/2_**  **(h)** | **T_max_**  **(h)** | **C_max_**  **(μg/ml)** | **MRT**  **(h)** | **CL/F**  **(ml/h/kg)** | **AUC_0-t_**  **(mg·h/ml)** |
| --- | --- | --- | --- | --- | --- | --- |
| **A1** | **440 ± 62** | **96 ± 0** | **13.1 ± 0.2** | **657 ± 82** | **0.11 ± 0.01** | **7.8 ± 0.3** |
| **B1** | **345 ± 91** | **96 ± 0** | **12.0 ± 0.1** | **547 ± 118** | **0.13 ± 0.02** | **7.2 ± 0.7** |
| **C1** | **327 ± 10** | **132 ± 36** | **7.9 ± 0.6** | **514 ± 44** | **0.21 ± 0.02** | **4.4 ± 0.7** |
| **D1** | **322 ± 10** | **96 ± 0** | **9.3 ± 0.4** | **485 ± 25** | **0.21 ± 0.00** | **4.6 ± 0.1** |
| **1 MPK Avg** | **359 ± 28** | **105 ± 9** | **10.6 ± 1.2** | **551 ± 38** | **0.17 ± 0.03** | **6.0 ± 0.9** |
|  |  |  |  |  |  |  |
| **A2** | **369 ± 27** | **120 ± 24** | **10.1 ± 0.8** | **562 ± 39** | **0.16 ± 0.02** | **5.8 ± 0.7** |
| **B2** | **435 ± 46** | **168 ± 0** | **13.7 ± 0.8** | **665 ± 63** | **0.10 ± 0.00** | **8.9 ± 0.0** |
| **C2** | **292 ± 9** | **96 ± 0** | **10.4 ± 1.5** | **419 ± 32** | **0.23 ± 0.05** | **4.3 ± 0.9** |
| **D2** | **314 ± 58** | **96 ± 0** | **8.7 ± 0.9** | **485 ± 93** | **0.22 ± 0.06** | **4.7 ± 1.1** |
| **1 MPK Avg** | **353 ± 32** | **120 ± 17** | **10.7 ± 1.1** | **533 ± 53** | **0.18 ± 0.03** | **5.9 ± 1.0** |
|  |  |  |  |  |  |  |
| **A1** | **502 ± 12** | **96 ± 0** | **68.2 ± 2.5** | **740 ± 18** | **0.10 ± 0.00** | **43.6 ± 0.6** |
| **B1** | **314 ± 6** | **48 ± 0** | **72.4 ± 1.4** | **463 ± 3** | **0.14 ± 0.00** | **33.9 ± 0.0** |
| **C1** | **265 ± 2** | **132 ± 36** | **38.5 ± 0.8** | **424 ± 20** | **0.28 ± 0.03** | **17.7 ± 1.9** |
| **D1** | **361 ± 8** | **168 ± 0** | **40.2 ± 1.1** | **554 ± 14** | **0.20 ± 0.01** | **23.5 ± 1.1** |
| **5 MPK Avg** | **361 ± 51** | **111 ± 26** | **54.8 ± 9.0** | **545 ± 70** | **0.18 ± 0.04** | **29.7 ± 5.7** |
|  |  |  |  |  |  |  |
| **A2** | **398 ± 47** | **96 ± 0** | **37.8 ± 1.8** | **601 ± 67** | **0.21 ± 0.02** | **22.0 ± 1.6** |
| **B2** | **434 ± 5** | **96 ± 0** | **68.0 ± 1.4** | **660 ± 5** | **0.10 ± 0.00** | **44.9 ± 1.2** |
| **C2** | **264 ± 10** | **72 ± 24** | **46.1 ± 0.9** | **399 ± 4** | **0.23 ± 0.01** | **21.0 ± 1.1** |
| **D2** | **413 ± 38** | **36 ± 12** | **51.5 ± 9.1** | **592 ± 48** | **0.17 ± 0.01** | **25.9 ± 2.4** |
| **5 MPK Avg** | **377 ± 38** | **75 ± 14** | **50.9 ± 6.4** | **563 ± 57** | **0.18 ± 0.03** | **28.5 ± 5.6** |
