# Supplementary material for: Pharmacokinetic comparison of a diverse panel of non-targeting human antibodies as matched IgG1 and IgG2 isotypes in rodents and non-human primates
Source: PLoS One. 2019 May 23;14(5):e0217061. doi: 10.1371/journal.pone.0217061 (PMC6533040; doi:10.1371/journal.pone.0217061)
Supplement: S4 Table — Differential scanning calorimetry (DSC) was performed on a Malvern MicroCal VP-Capillary DSC. See Fig 8. The following parameters were used: scanning range: 10–100°C; scanning rate: 1°C/min; pre-scan thermostat 15 min. Typically 400 μL of 1 mg/mL sample is consumed for each analysis. Data was processed in Origin 7 software. (DOCX) [file pone.0217061.s011.docx]

| **Ab** | **T_m1_**  **(°C)** | **T_m2_**  **(°C)** | **T_m3_**  **(°C)** |
| --- | --- | --- | --- |
| **A1** | **70.8** | **78.4** | **83.4** |
| **B1** | **71.1** | **79.0** | **82.3** |
| **C1** | **70.7** | **84.5** | **84.5** |
| **D1** | **71.6** | **79.0** | **82.3** |
|  |  |  |  |
| **A2** | **69.6** | **75.7** | **82.7** |
| **B2** | **70.7** | **80.6** | **82.3** |
| **C2** | **70.7** | **78.9** | **84.5** |
| **D2*** | **71.6** | **78.9** | **82.3** |
